# Acceptorless dehydrogenation of small molecules through cooperative base metal catalysis

**DOI:** 10.1038/ncomms10093

**Published:** 2015-12-11

**Authors:** Julian G. West, David Huang, Erik J. Sorensen

**Affiliations:** 1 Department of Chemistry, Princeton University, Princeton, New Jersey 08544, USA

## Abstract

The dehydrogenation of unactivated alkanes is an important transformation both in industrial and biological systems. Recent efforts towards this reaction have revolved around high temperature, organometallic C–H activation by noble metal catalysts that produce alkenes and hydrogen gas as the sole products. Conversely, natural desaturase systems proceed through stepwise hydrogen atom transfer at physiological temperature; however, these transformations require a terminal oxidant. Here we show combining tetra-*n*-butylammonium decatungstate (TBADT) and cobaloxime pyridine chloride (COPC) can catalytically dehydrogenate unactivated alkanes and alcohols under near-UV irradiation at room temperature with hydrogen as the sole by-product. This noble metal-free process follows a nature-inspired pathway of high- and low-energy hydrogen atom abstractions. The hydrogen evolution ability of cobaloximes is leveraged to render the system catalytic, with cooperative turnover numbers up to 48 and yields up to 83%. Our results demonstrate how cooperative base metal catalysis can achieve transformations previously restricted to precious metal catalysts.

## Abstract (web summary)

Acceptorless dehydrogenation of simple alkanes and alcohols provides useful functionality and typically requires precious metal catalysts. Here, the authors dehydrogenate unactivated alkanes and alcohols at room temperature using earth-abundant metals with hydrogen as the sole by-product.

## Graphical abstract

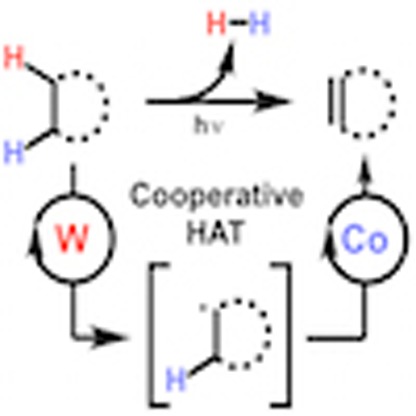

Alkenes have long been recognized as powerful footholds^1^ in syntheses of complex natural products and pharmaceutical agents, with many targets containing the motif itself^2^. One strategy that has been employed by both chemists and biological systems to introduce this essential functionality is the dehydrogenation of saturated alkyl motifs, a transformation that allows for almost unlimited flexibility in the placement of this unsaturation. Hydrogen production also remains an important challenge to this day^3^, with the development of acceptorless (terminal oxidant free) methods possessing the added appeal of achieving this goal. While the literature pertaining to catalytic dehydrogenation is replete with creative advances^1^,^2^, we recognized a gap in current knowledge: acceptorless and noble metal-free methods have been developed, however, no system combining these two desirable properties was known.

Many efforts to achieve dehydrogenations of alkanes have drawn inspiration from the seminal reports by Bergman^4^ and Graham^5^ on the oxidative addition of low-valent iridium complexes into C–H bonds. Further development of this organometallic reaction manifold by Felkin^6^, Crabtree^7^, Goldman^8^,^9^ and others has resulted in the development of noble metal catalysts that generate alkenes from alkanes through a coupling of this initial step with subsequent ß-hydride elimination and reductive elimination to liberate hydrogen as the sole by-product (Fig. 1a)^10^. These important transformations display high turnover numbers and function without a terminal oxidant, although they also require expensive noble metal catalysts and high temperatures to be efficient. Photocatalytic systems based on iridium^7^ and rhodium^11^,^12^,^13^ are known and can be highly efficient; however, these processes still adhere to fundamentally organometallic mechanisms and typically have high-temperature requirements.

Nature employs an approach complementary to the organometallic activation strategy. Careful study of natural, iron-based desaturase systems has revealed that the first step involves a hydrogen atom transfer (HAT) from the substrate by a high-valent oxo species^14^. Following this rate-determining step, either a second, rapid HAT (pictured, Fig. 1b) or stepwise electron transfer-proton transfer to the catalyst generates the desired olefin and a reduced equivalent of catalyst. Stoichiometric experiments by Breslow *et al.*^15^ showed that sequential HAT is a viable desaturation mechanism in synthetic contexts and, subsequently, bio-inspired catalytic systems have been developed^16^. This enzymatic HAT strategy proceeds at low temperature using an earth-abundant metal; however, it is incapable of hydrogen-gas production and requires a stoichiometric oxidant to proceed.

We envisioned that a third mechanistic scenario (Fig. 1c), maintaining both the mild temperature requirements of enzymatic systems and the oxidant-free operation/hydrogen production ability of the noble metal strategy, should be attainable through judicious design of the reaction. Additionally, the relative rarity of noble metals led us to focus our thinking on base metal catalysts that are easily accessible from commodity materials, an important stipulation and remaining challenge in catalysis. In this design exercise, we took two key lessons from the desaturase systems: the amenability of radical dehydrogenation to mild temperature regimes and the marked difference in C–H bond strength between an unactivated alkane and a C–H bond ß to an alkyl radical^17^. Combining these with an awareness of the expanded reactivity possibilities of using cooperative catalysis^18^, we considered whether two catalysts, one capable of activating a strong (∼100 kcal mol^–1^) C–H bond, (‘hard' HAT, cat A, Fig. 1c), and another capable of activating a weakened (<50 kcal mol^–1^) bond, (‘easy' HAT, cat B) could be combined. Following this cooperative HAT (cHAT) sequence, it was imagined that each catalyst could donate a hydrogen atom equivalent to liberate H_2_ and turnover the system.

Towards pursuing this reaction manifold, a survey of the radical HAT literature immediately revealed cobaloximes as easily synthesized base metal complexes with intriguing properties (Fig. 2). Indeed, it is known from both the radical polymerization^19^ and synthetic literature^20^ that cobaloximes can reversibly form olefins and cobalt hydrides^21^,^22^ (The formulation of this ‘hydride' is currently under debate in the literature; for recent discussions of this, please see refs 21, 22) in the presence of alkyl radicals (as required for cat B). Cobaloximes are also known in the context of hydrogen evolution^23^, with studies implicating this same hydride as a key intermediate in the catalytic cycle^24^. Taken together, this suggested to us that a HAT-hydrogen evolution cascade might be possible by leveraging both of these known reactivities. With a candidate for cat B found, we began our search for cat A.

Inspired by the success of metal-oxo compounds in enzymatic desaturation, our attention was immediately drawn to the polyoxometalate, tetra-*n-*butylammonium decatungstate (TBADT, Fig. 2), a versatile, easily synthesized compound which is able to effect HAT from unactivated alkanes upon irradiation by near-UV light (including solar irradiation)^25^. Photoredox reactivty has been a mainstay in cooperative catalysis and systems combining noble metal sensitizers and cobaloximes have been reported for water reduction^26^, suggesting that TBADT might be productively combined with cobaloxime pyridine chloride (COPC). Additionally, the inclusion of near-UV light revises the energetics of the process, making the otherwise endothermic acceptorless dehydrogenation exothermic (values shown for cyclooctane and the maximum absorbance of TBADT, Fig. 3). Hill *et al.*^27^ were able to achieve a related transformation, the production of non-thermodynamic olefins and alkane dimers, through HAT to photoexcited TBADT, followed by disproportionation and/or coupling of the intermediate organic radicals. Addition of platinum metal to this reaction rendered the dehydrogenation catalytic; however, the requirement of a heterogeneous, noble metal cocatalyst for this system could be viewed as a drawback, with the reliance on the disproportionation of alkyl radicals for olefin formation affording a disappointing product ratio of 1.3:1 olefin to dimer using cyclooctane. We reasoned that a pairing of TBADT with COPC might overcome these hurdles.

Our studies find that these catalysts can be productively combined to enable the acceptorless dehydrogenation of both alkanes and alcohols. Dehydrogenation product selectivities exhibit an interesting divergence from noble metal systems and radical coupling products are not observed. The reaction appears to proceed through the designed cHAT mechanism and presents a unique approach to dehydrogenative processes.

## Results

### Dehydrogenation of alkanes

Subjecting an acetonitrile solution of cyclooctane to near-UV irradiation in the presence of 1.5 mol% TBADT led to the formation of a trace (substoichiometric with respect to TBADT) amount of cyclooctene (Table 1, entry 1), a result in agreement with that of Hill *et al.*^27^. A similar treatment of a solution containing 0.75 mol% COPC and no TBADT led to no change to the starting alkane both with and without light (Table 1, entries 3 and 4). To our gratification, irradiation of a solution containing cyclooctane, TBADT and COPC produced cyclooctene in 23% yield, as well as hydrogen gas, with ∼15 cooperative turnovers (Table 1, entry 5).

Further exploration of the reaction conditions found that TBADT, cobaloxime and light are all required for catalysis. The reaction may also be driven by solar irradiation, although an incandescent lamp was ineffective for reactivity. Increasing the catalyst loadings did not significantly increase the efficiency of the process and also led to the production of byproducts, 1-butene and tributylamine, from the known decomposition of the tetra-*n*-butyl ammonium cations^28^. Interestingly, reducing both catalyst loadings to sub-mol% values did not negatively affect the progress of the reaction, with cooperative turnover numbers of up to 48 able to be achieved (Table 1, entry 9). Cyclopentane was successfully dehydrogenated to cyclopentene and cyclopentadiene, while cyclohexane was predominantly dehydrogenated to cyclohexene (Table 2, entries 2 and 3). The selectivity for cyclohexene over the aromatic benzene is striking in comparison to noble-metal systems, with cyclohexene itself being largely inert to the reaction conditions (Table 2, entry 4). The most contrasting result comes from ethyl isovalerate: with this substrate, only the skipped enone product was formed (Table 2, entry 5). As organometallic dehydrogenations proceed through isomerization-promoting metal-hydride intermediates, the production of this non-thermodynamic product selectively is a unique advantage of this catalyst system. Additionally, the tolerance of this process for such a chemically sensitive compound suggests that other functionalized and intricate substrates might also be amenable to a future iteration of this system. The relatively poor efficiency of these substrates compared with cyclooctane is unsurprising, as it is known that cyclooctane is a particularly well-behaved dehydrogenation substrate^8^,^27^.

### Dehydrogenation of alcohols

We hypothesized that a limiting factor of the reaction's progress may be the inherent endothermicity of acceptorless dehydrogenation, with cyclooctane being most successful due to its low heat of hydrogenation (–23 kcal mol^–1^, Fig. 3)^29^. The high strength of the carbonyl *π*-bond relative to that of alkenes suggested that alcohols might exhibit a stronger driving force for dehydrogenation than alkanes, potentially making these more well behaved for this catalyst system and proposed mechanism (Fig. 4). The relative success of organometallic approaches^10^,^30^,^31^ to this reaction class, as compared with alkane dehydrogenation, further drew us to studying it under our conditions. Initial experiments with 2-propanol (heat of hydrogenation – 13 kcal mol^–1^)^32^ confirmed this thinking, with yields of up to 63% being achievable through simple variation of reaction conditions (Table 3, entry 1 and Supplementary Table 2).

Additionally, the presence of both catalysts and light-proved essential for reactivity as was observed for alkanes. Extending these conditions to other secondary alcohols lead to good yields of the product ketones with the concomitant evolution of hydrogen gas, with the highest yield observed being 83% using 1-phenylethanol (Table 3). Attempts to dehydrogenate primary alcohols, such as 1-butanol, led to trace conversion (Table 3, entry 4). A potential cause of this observation could be competitive, unproductive abstraction of the activated aldehyde C–H bond of the products, a known mode of reactivity for TBADT (ref. 33).

### Effect of cobalt ligand environment

Having demonstrated the mutual compatibility of TBADT and COPC in mild dehydrogenations of selected hydrocarbons and alcohols, we determined if the identity of the cobalt species is essential for reactivity. It is known from the polymerization literature^34^ that efficient HAT requires a planar, κ-4 ligand (such as the dmgH motif). Moreover, glyoximes are known to be a privileged ligand framework for hydrogen evolution reactions^23^. The substitution of COPC by either cobalt(II) acetate or cobalt(II) acetoacetonate in the cyclooctane reaction led to results identical to that of having no cobalt cocatalyst, while the inclusion of cobaloxime boron fluoride, a related, Co(II) complex, led to comparable results to those achieved with COPC (Supplementary Fig. 1). These experiments suggest that the identity of the cobalt ligand environment is relevant to the reaction, as all tested complexes are soluble in acetonitrile at the concentrations examined (1 mol%). While alkene was only produced in super-stoichiometric quantities in the presence of cobaloxime cocatalysts, conversion to undesired products was observed in all irradiated reactions containing TBADT.

### Mechanistic considerations

**Figure 1:**
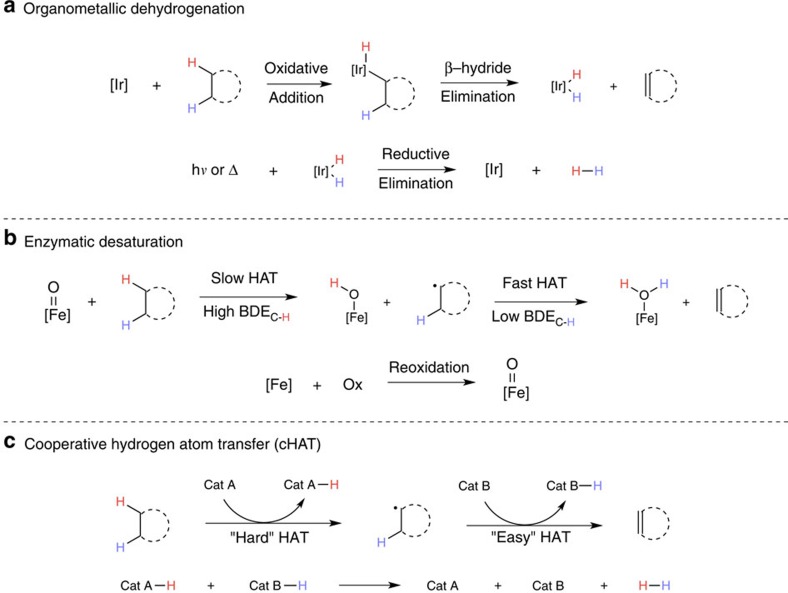
Strategies for the dehydrogenation of alkanes. (**a**) acceptorless and organometallic (**b**) oxidative and enzymatic. We envisioned a third process (**c**) whereby the dehydrogenation proceeds under mild conditions as in **b** yet still evolves hydrogen as in **a**.

**Figure 3:**
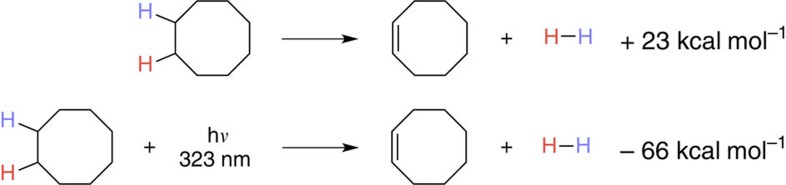
Energetics of acceptorless dehydrogenation with and without near-ultraviolet irradiation. An absorbance of 323 nm was chosen for the calculation as it is the absorbance maximum of TBADT.

**Figure 4:**
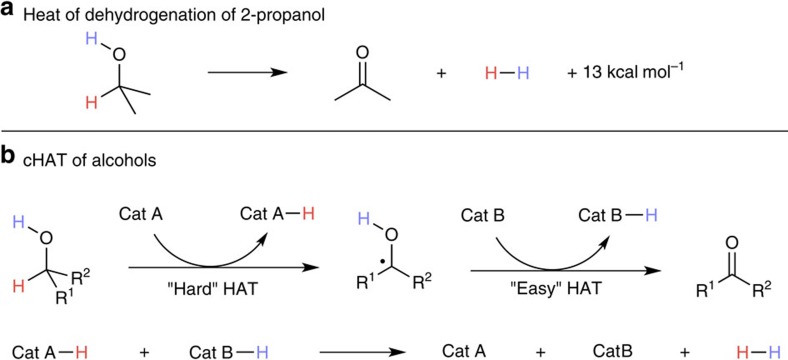
Dehydrogenation of alcohols. (**a**) Heat of dehydrogenation of 2-propanol. (**b**) A proposed sequence for the dehydrogenation of alcohols through cHAT.

**Figure 5:**
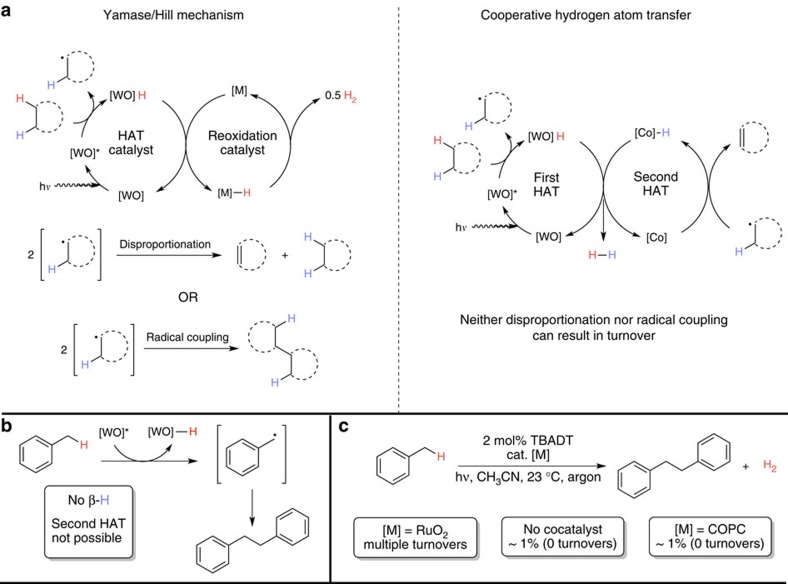
Potential mechanisms for the TBADT/COPC-catalyzed dehydrogenation of alkanes. (**a**) the process might proceed through either the Yamase/Hill mechanism, with final products due to radical disproportionation or coupling, or the expected cHAT mechanism. **(b)** The dimerization of toluene can only be catalytic via the Yamase/Hill mechanism. **(c)** The dimerization of toluene is not catalytic using the TBADT/COPC catalyst system, suggesting that cHAT might be operative.

Following these explorations, we returned to the question of reaction mechanism. Based on the design outlined in Fig. 1c, we envisioned a dehydrogenation through successive HATs to different catalysts (cHAT, Fig. 5a). An important aspect of this mechanistic scenario is that turnover is only possible if each catalyst accepts a hydrogen atom from the substrate; if disproportionation or radical coupling occurs, a cHAT process will stall.

An alternative mechanism would invoke the cobaloxime cocatalyst as a reoxidant for the quenched decatungstate species [WO]–H (Yamase/Hill mechanism, Fig. 5a), the operative mechanism for TBADT/heterogenous noble metal systems^28^. While we already had compelling evidence for a mechanistic divergence with the observed product ratios (1.3:1 cyclooctene/dicyclooctane for TBADT/Pt and no dicyclooctene observed under TBADT/COPC catalysis) and hydrogen stoichiometry (1:1 alkene/hydrogen), we set out to probe the potential relevance of this mechanism by selecting a reaction that can only advance via coupling, an example being the dimerization of toluene (Fig. 5b). This reaction is known using a combination of TBADT and RuO_2_ (ref. 35). In this study, when toluene was exposed to 2 mol% of decatungstate under UV irradiation with and without cobaloxime cocatalyst, an identical, substoichiometric formation of bibenzyl was observed, even after prolonged (>72 h) irradiation. This experiment suggests that HAT from [WO]–H to the cobaloxime cocatalyst is not a dominant process. Additionally, continuous photolysis ultraviolet-visible experiments of both the cyclooctane and toluene reactions showed an accumulation of the efficient HAT species [Co^II^] when COPC was used as the catalyst (Supplementary Figs. 3–30). The concentration of this species remains significant throughout the course of the reactions, meaning that the rapid, second HAT of cHAT should occur readily in cases where it is possible. In both cases, it was observed that reduced TBADT accumulates in the presence of Co^II^; this is also inconsistent with the cobaloxime serving as a reoxidant for the reduced TBADT.

## Discussion

Collectively, these experiments exemplify how inspiration from both synthetic and biological systems can guide the design of a mild, new method to dehydrogenate certain saturated hydrocarbons and alcohols with evolution of hydrogen gas. Our observations are consistent with a dehydrogenation process featuring a cHAT pathway, a mechanism that has no near neighbour in the catalysis literature. The combination of two base metal catalysts is shown to be a powerful method to achieve new transformations, and we expect many future developments using this strategy.

## Methods

An oven-dried 17 × 60 mm (8 ml) borosilicate vial was equipped with a magnetic stir bar, TBADT (0.004–0.03 equiv), and COPC (0.004–0.015 equiv). Following the addition of methyl acetate (3–4 drops) and CD3CN (1.1 ml, ∼0.65 M) the vial was fitted with a silicone septa screw cap and sparged with argon for 10 min. The substrate (0.65 mmol, 1 equiv, previously sparged with Ar) was added by syringe and stirred vigorously away from ultraviolet light sources. A *t*=0 aliquot was then removed using a syringe for calibrating the internal standard and the vial was sealed with parafilm and placed ∼5 cm from a 26-W compact fluorescent light bulb. Successful reactions changed colour from clear, pale yellow solutions to dark blue solutions within 1 h of the start of irradiation. After 48 h, hydrogen production was confirmed by headspace gas chromatography-thermal conductivity detector (GC/TCD) analysis. The alkene production was then assayed against the *t*=0 aliquot. If headspace analysis was not required, it is prudent to note that breakage of the vial seal was accompanied by the release of pressure, corresponding to the generated hydrogen.

Alternatively, if hydrogen production was to be quantified, the reaction was set-up analogously to above save no internal standard was included and no aliquots were removed from the reaction mixture. After the specified time of irradiation, the volume of evolved gas was measured through the equilibration of the reaction headspace pressure with atmospheric pressure using a gas syringe. Following the measurement of hydrogen, the amount of alkene was determined using 1,3,5-trimethoxybenzene as an internal standard.

See Supplementary Methods for further details.

## Additional information

**How to cite this article:** West, J. G. *et al.* Acceptorless dehydrogenation of small molecules through cooperative base metal catalysis. *Nat. Commun.* 6:10093 doi: 10.1038/ncomms10093 (2015).

## Supplementary Material

Supplementary Information Supplementary Figures 1-32, Supplementary Tables 1-4, Supplementary Discussion, Supplementary Methods and Supplementary References

## Figures and Tables

**Figure 2:**
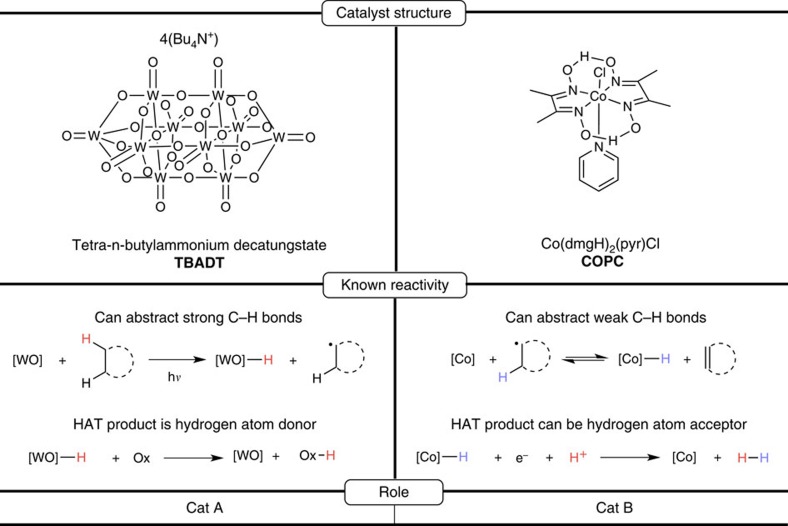
Catalyst selection for cHAT. TBADT and COPC are natural choices for cat A and cat B, respectively, due to their known reactivities.

**Table 1:**
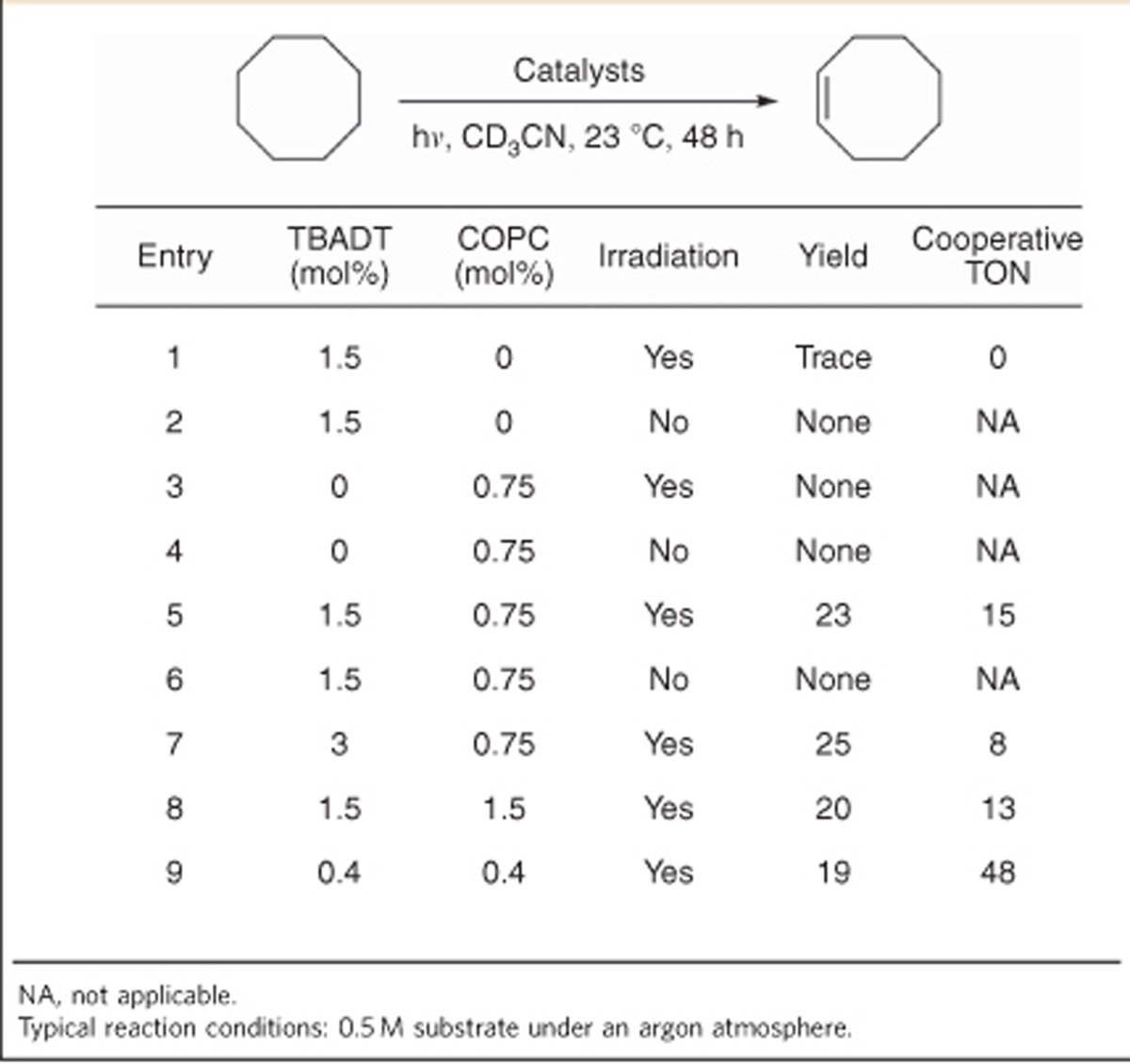
Development of a dehydrogenation method using cooperative base metal catalysis.

**Table 2:**
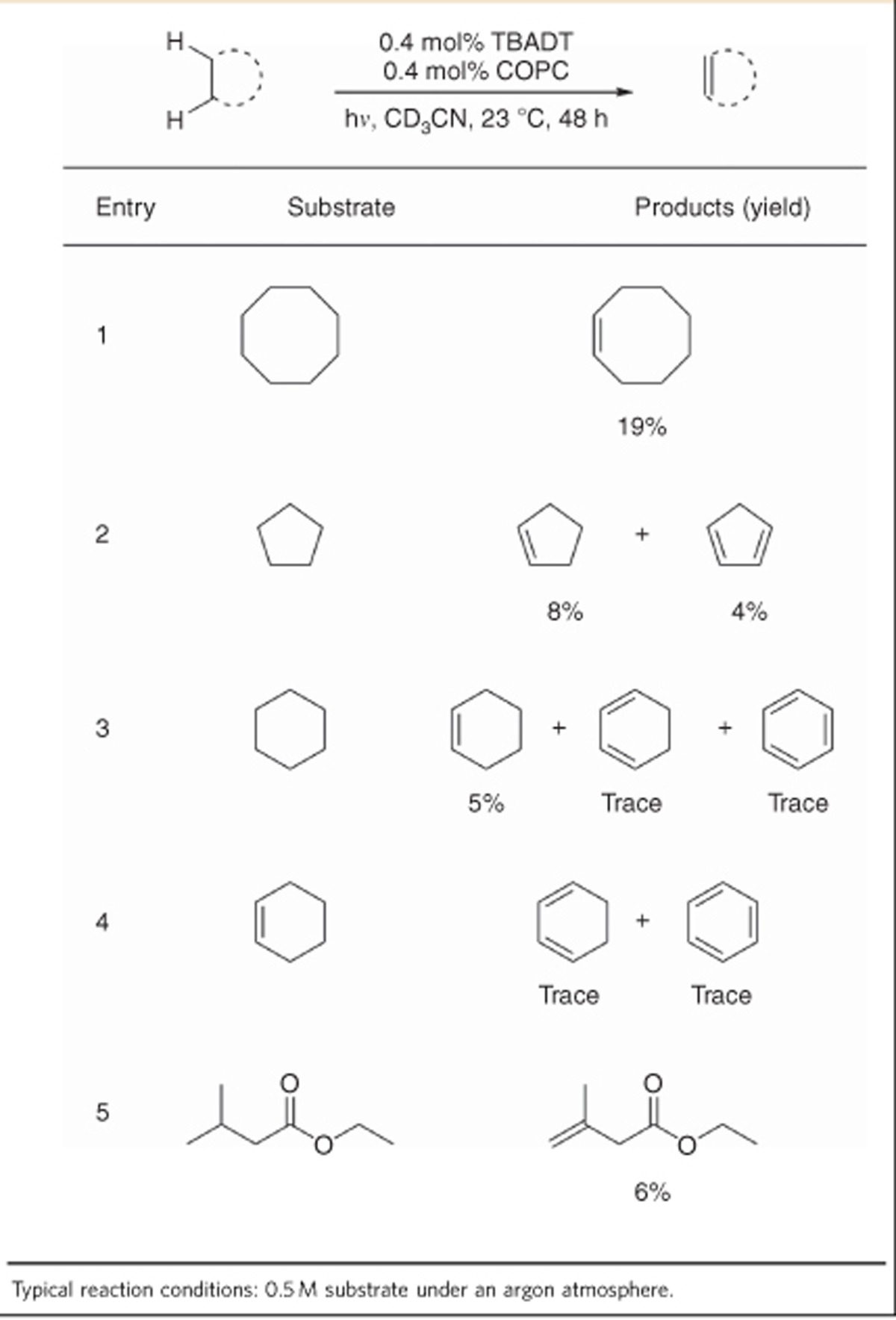
Dehydrogenation of alkanes through cooperative catalysis.

**Table 3:**
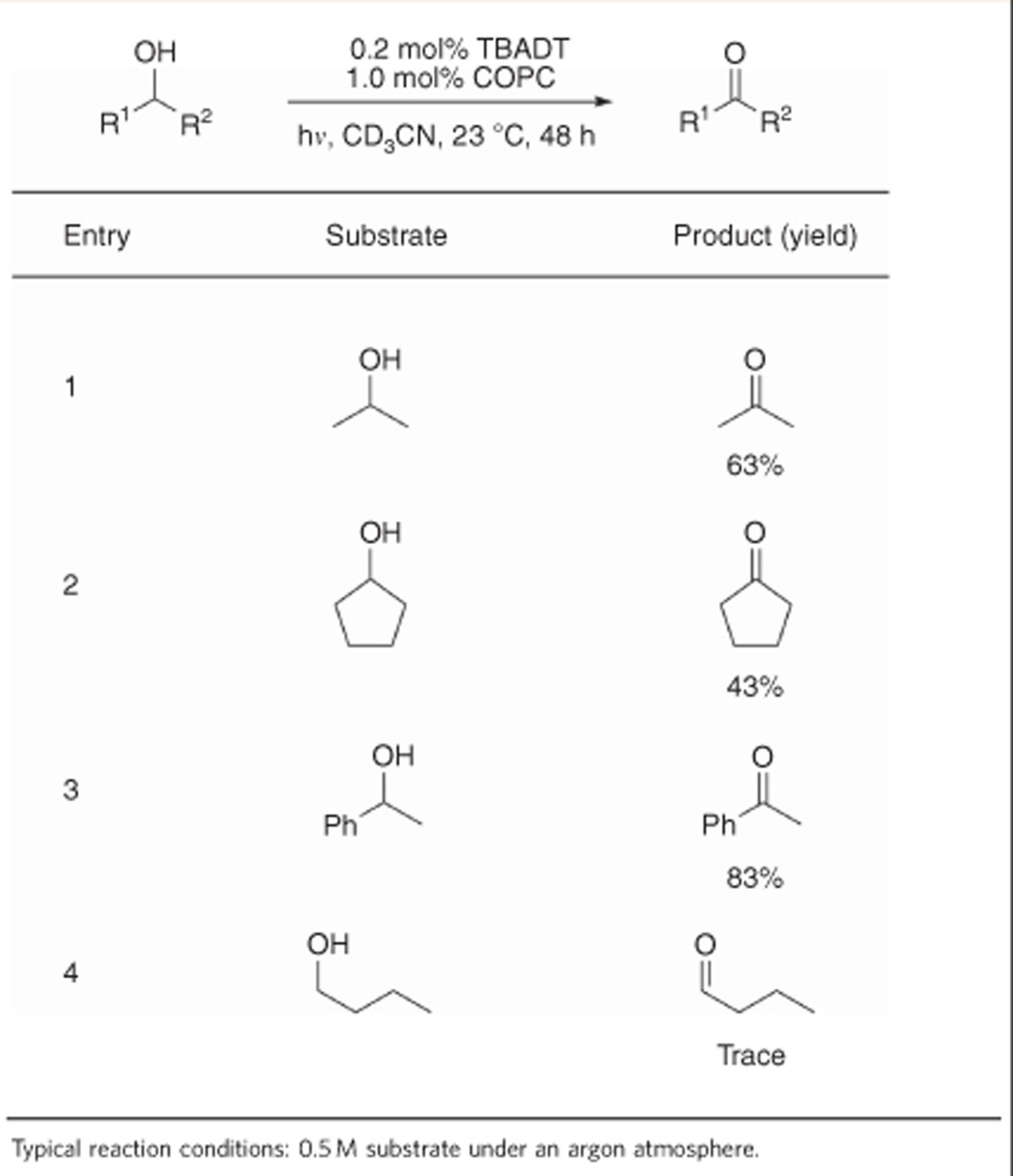
Dehydrogenation of alcohols through cooperative catalysis.
