# Supplementary material for: Acceptorless dehydrogenation of small molecules through cooperative base metal catalysis
Source: Nat Commun. 2015 Dec 11;6:10093. doi: 10.1038/ncomms10093 (PMC4682047; doi:10.1038/ncomms10093)
Supplement: Supplementary Information — Supplementary Figures 1-32, Supplementary Tables 1-4, Supplementary Discussion, Supplementary Methods and Supplementary References [file ncomms10093-s1.pdf]

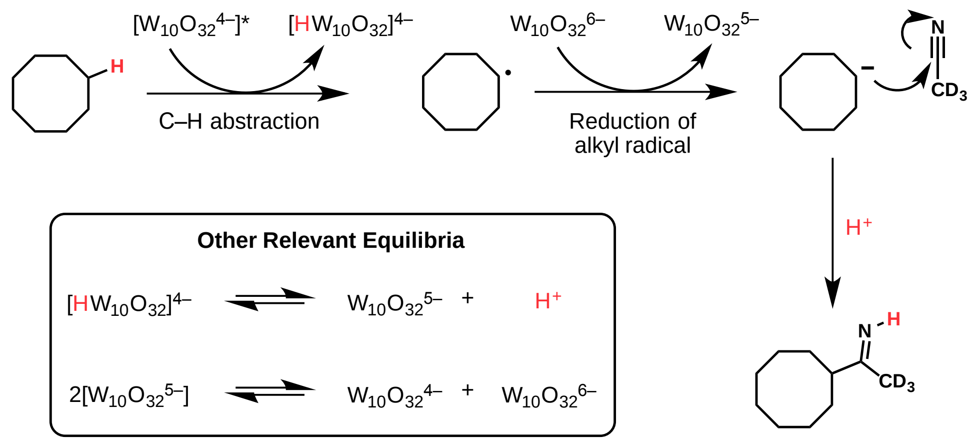

Supplementary Figure 1. A possible side reaction responsible for the consumption of starting material both in the presence and absence of cobalt cocatalyst

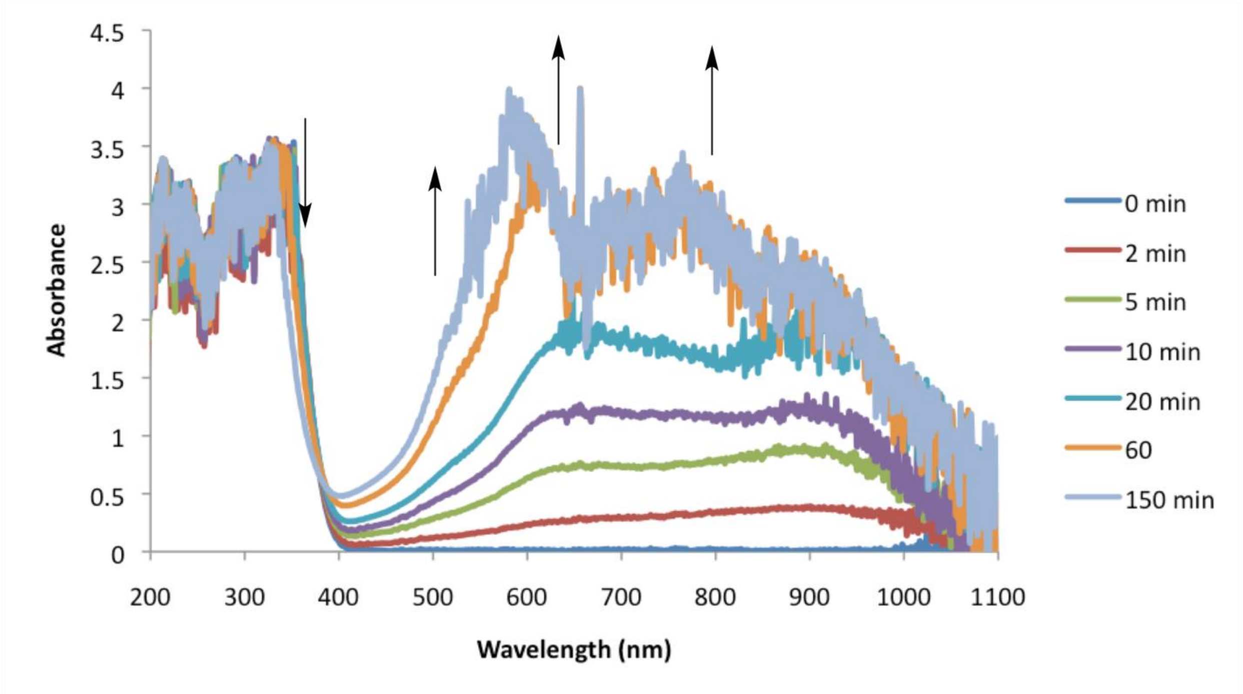

Supplementary Figure 2. Absorbance spectrum of continuous photolysis of a solution of Cyclooctane and 0.4mol% TBADT

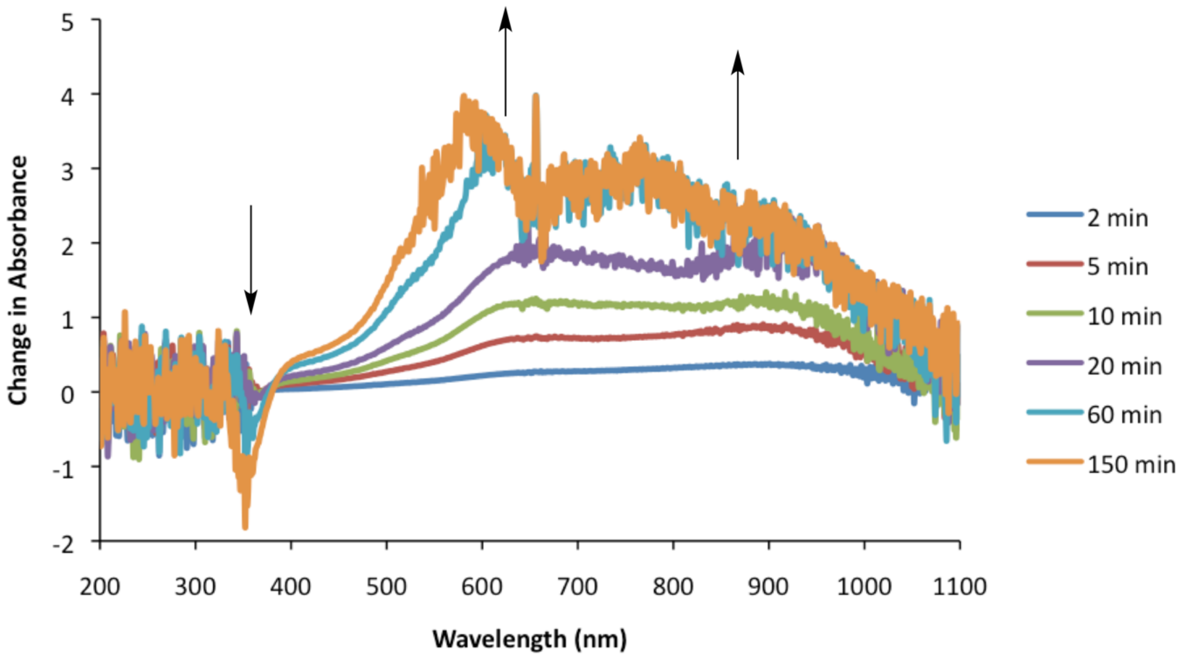

Supplementary Figure 3. Difference absorbance spectrum from the continuous photolysis of a solution of Cyclooctane and 0.4mol% TBADT

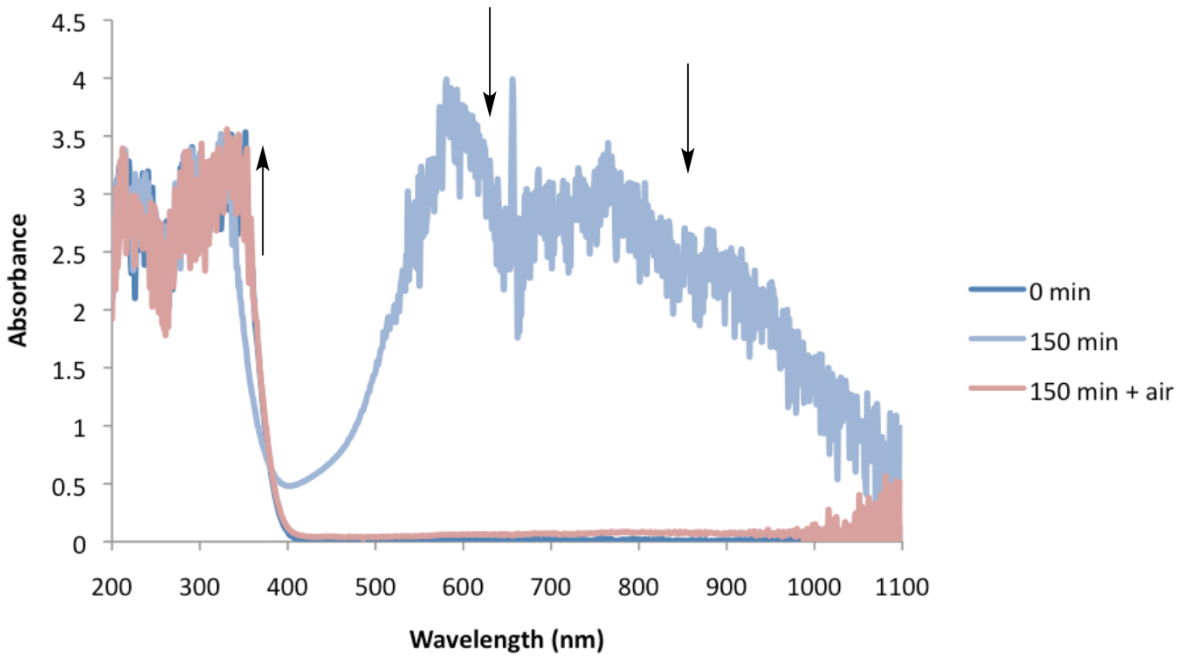

Supplementary Figure 4. Effect of oxygenation of continuously irradiated solution of cyclooctane and 0.4 mol% TBADT, absorbance spectrum

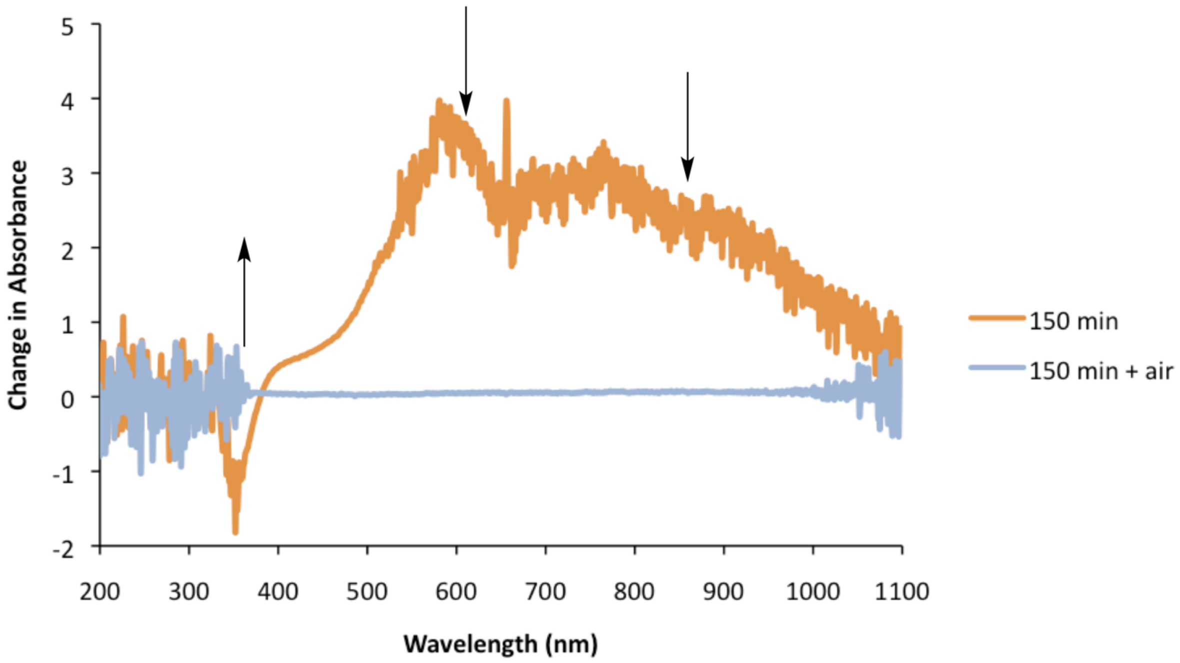

Supplementary Figure 5. Effect of oxygenation of continuously irradiated solution of cycloooctane and 0.4 mol% TBADT, difference absorbance spectrum

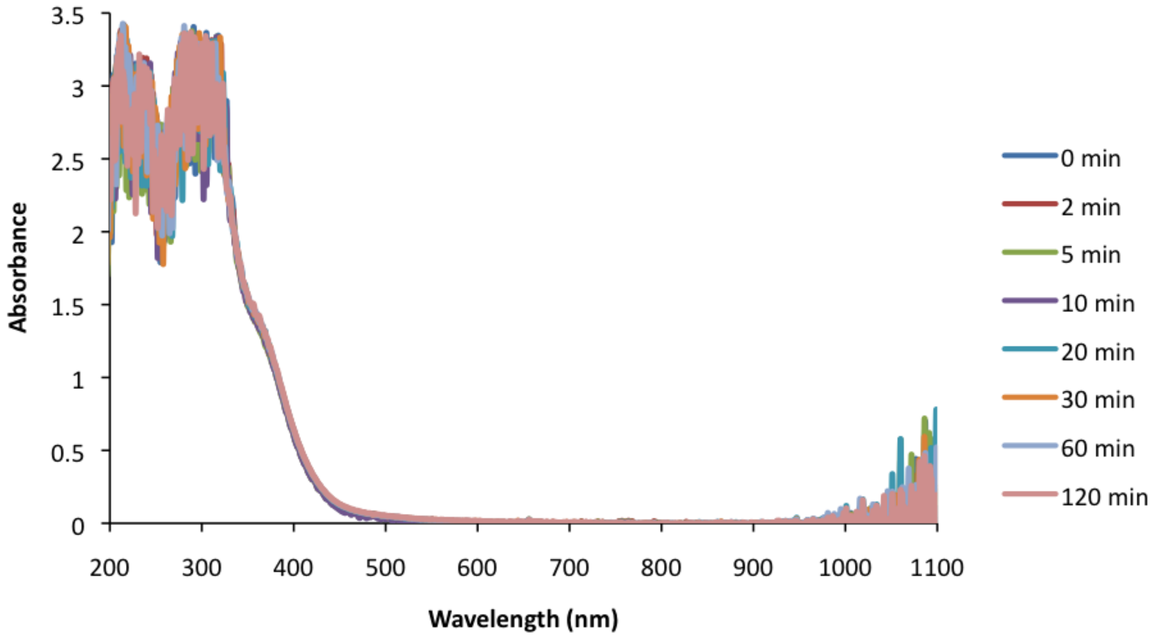

Supplementary Figure 6. Absorbance spectrum of continuous photolysis of a solution of Cyclooctane and 0.4mol% COPC

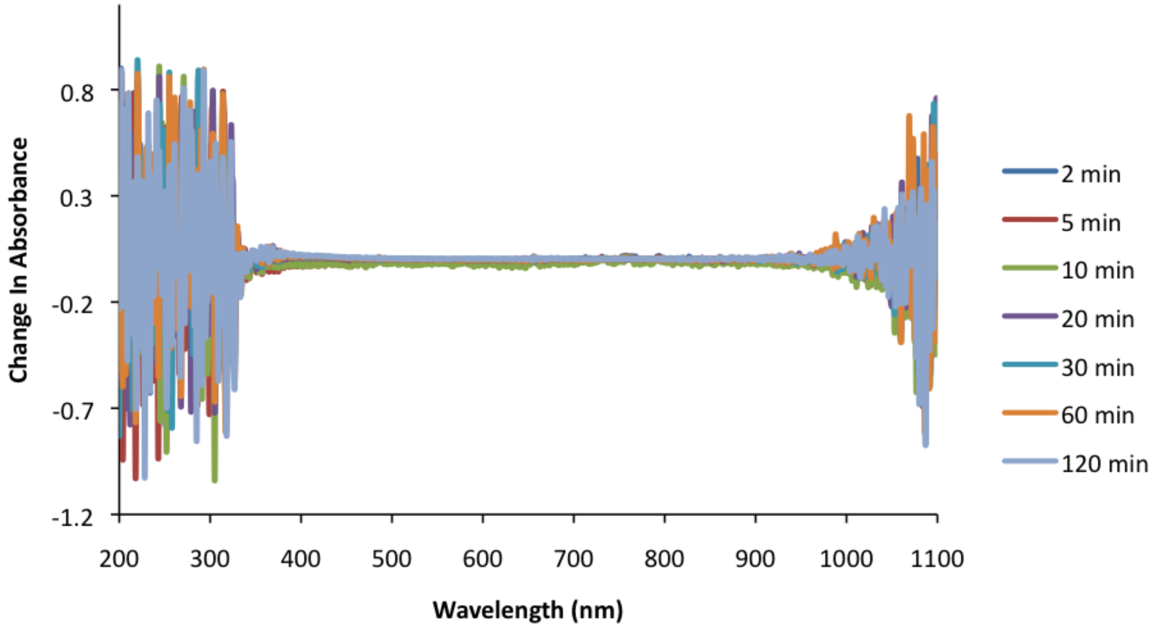

Supplementary Figure 7. Difference absorbance spectrum of continuous photolysis of a solution of Cyclooctane and 0.4mol% COPC

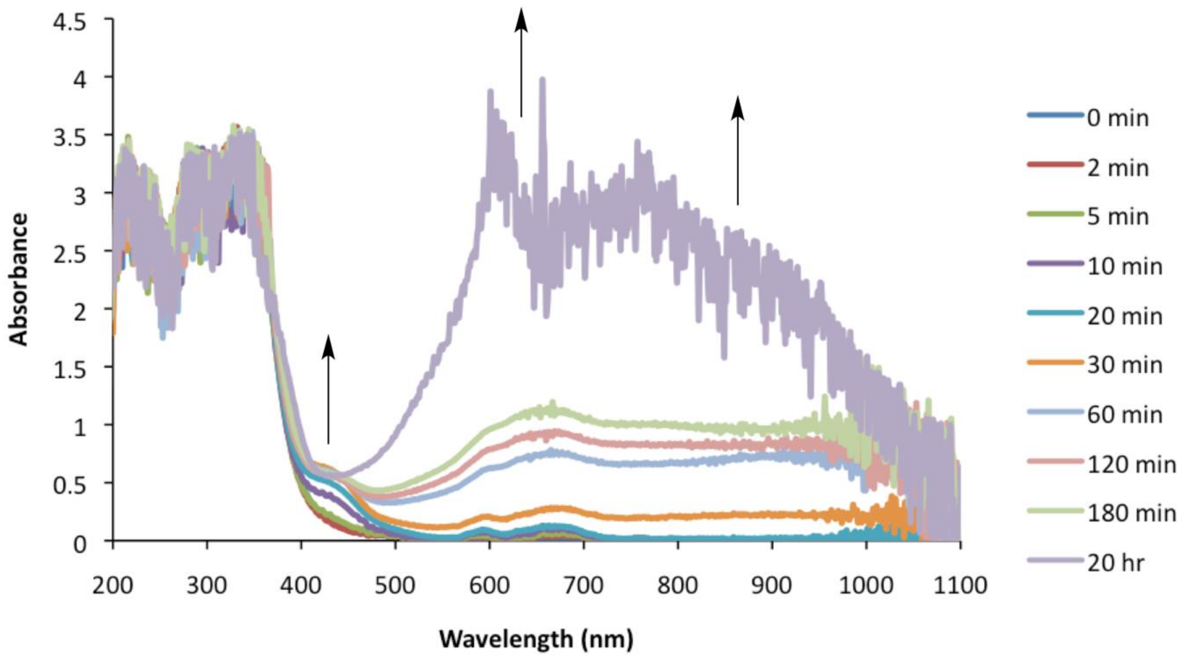

Supplementary Figure 8. Absorbance spectrum of continuous photolysis of a solution of Cyclooctane 0.4 mol% TBADT, and 0.4 mol% COPC

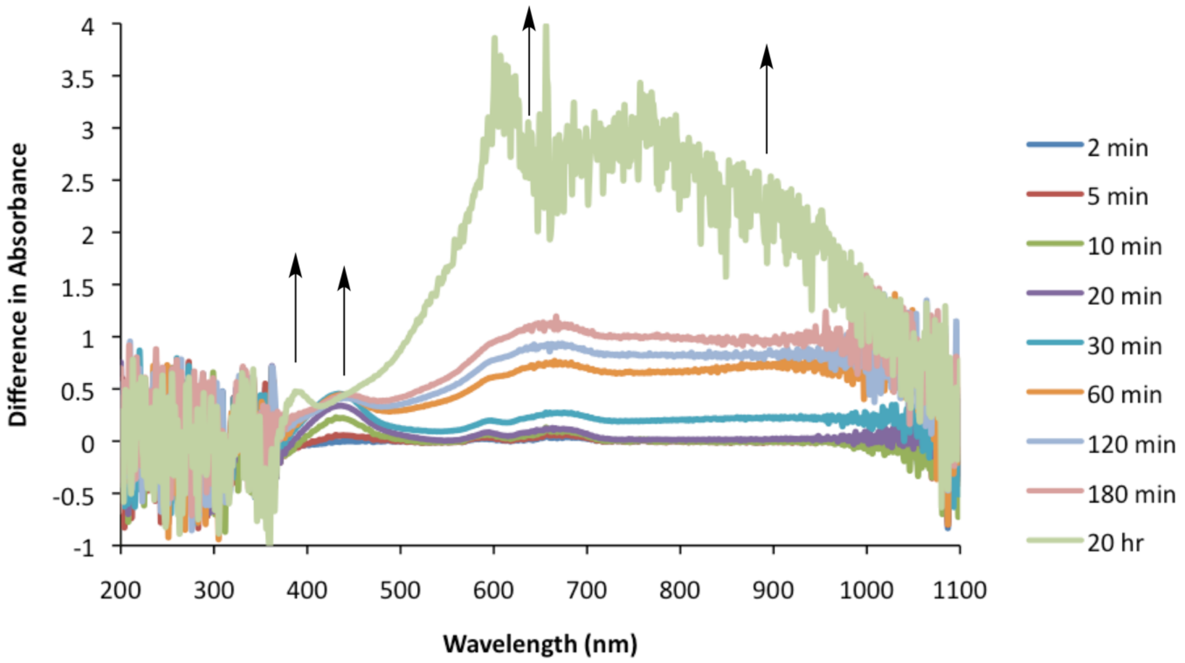

Supplementary Figure 9. Difference absorbance spectrum of continuous photolysis of a solution of Cyclooctane 0.4 mol% TBADT, and 0.4 mol% COPC

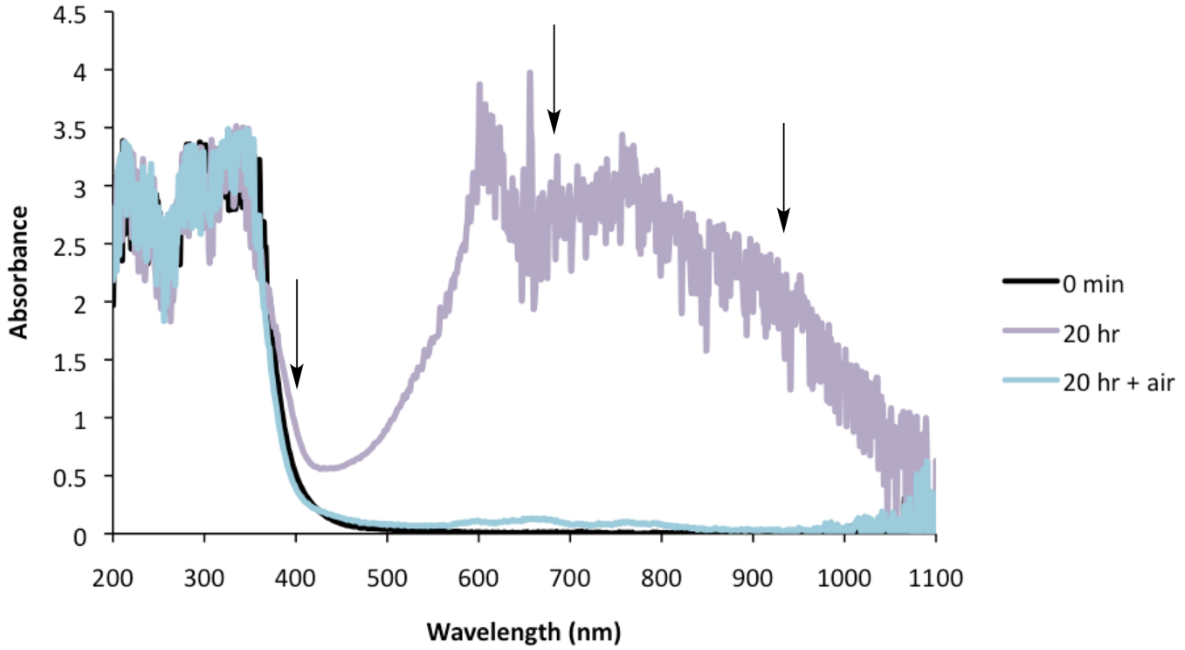

Supplementary Figure 10. Effect of oxygenation on the absorbance spectrum of continuous photolysis of a solution of Cyclooctane 0.4 mol% TBADT, and 0.4 mol% COPC, absorbance spectrum

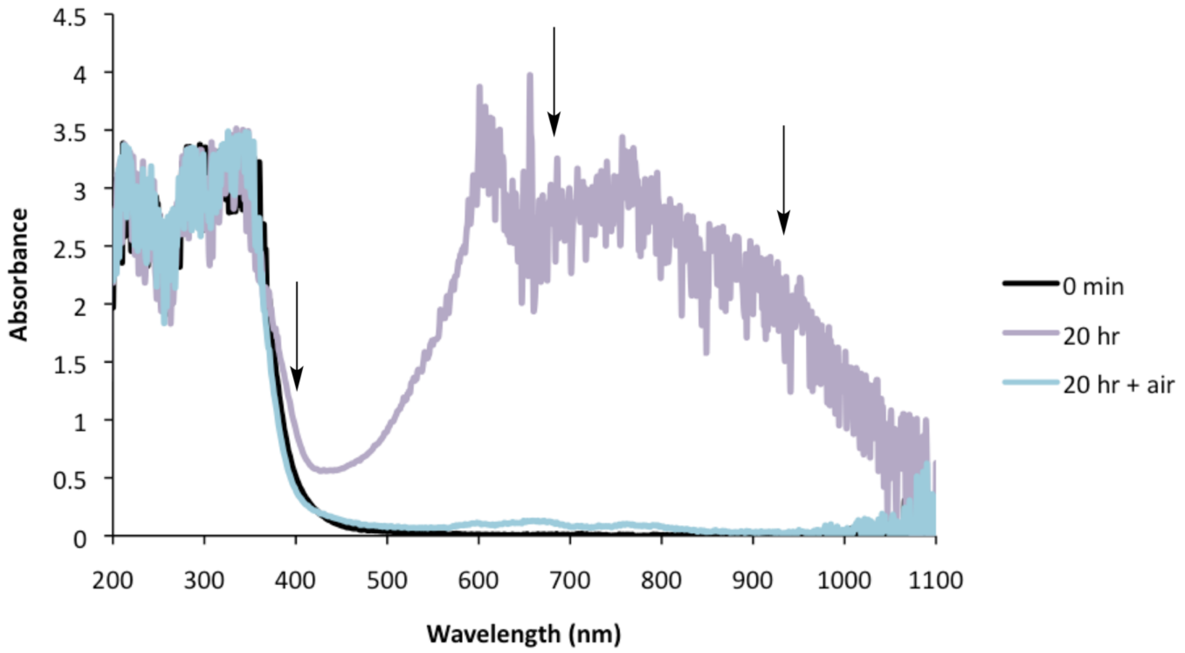

Supplementary Figure 11. Effect of oxygenation on the absorbance spectrum of continuous photolysis of a solution of Cyclooctane 0.4 mol% TBADT, and 0.4 mol% COPC, difference absorbance spectrum

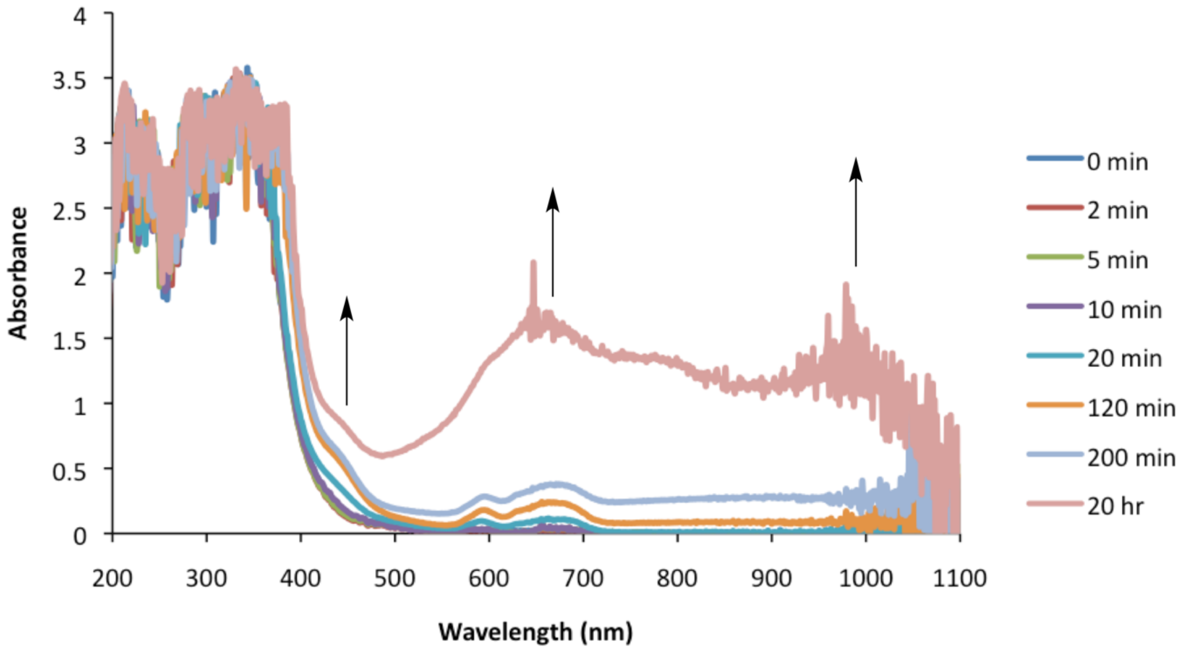

Supplementary Figure 12. Absorbance spectrum of continuous photolysis of a solution of toluene, 0.4 mol% TBADT, and 0.4 mol% COPC

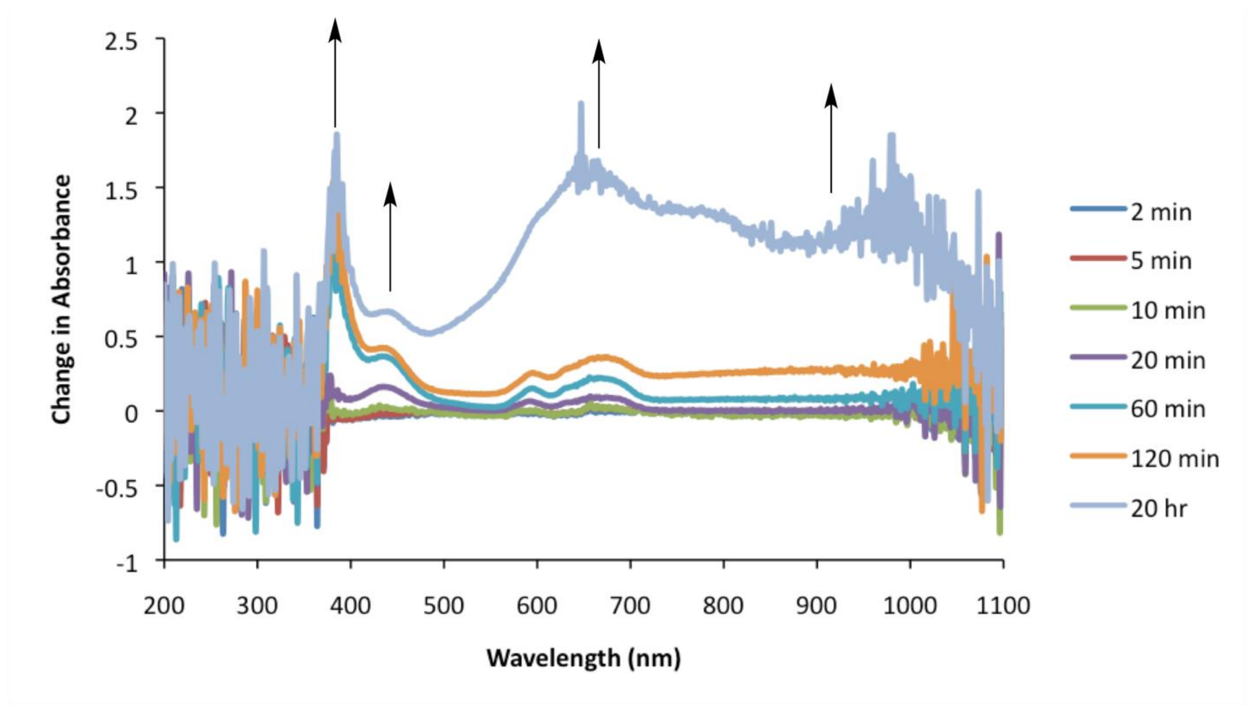

Supplementary Figure 13. Difference absorbance spectrum of continuous photolysis of a solution of toluene, 0.4 mol% TBADT, and 0.4 mol% COPC

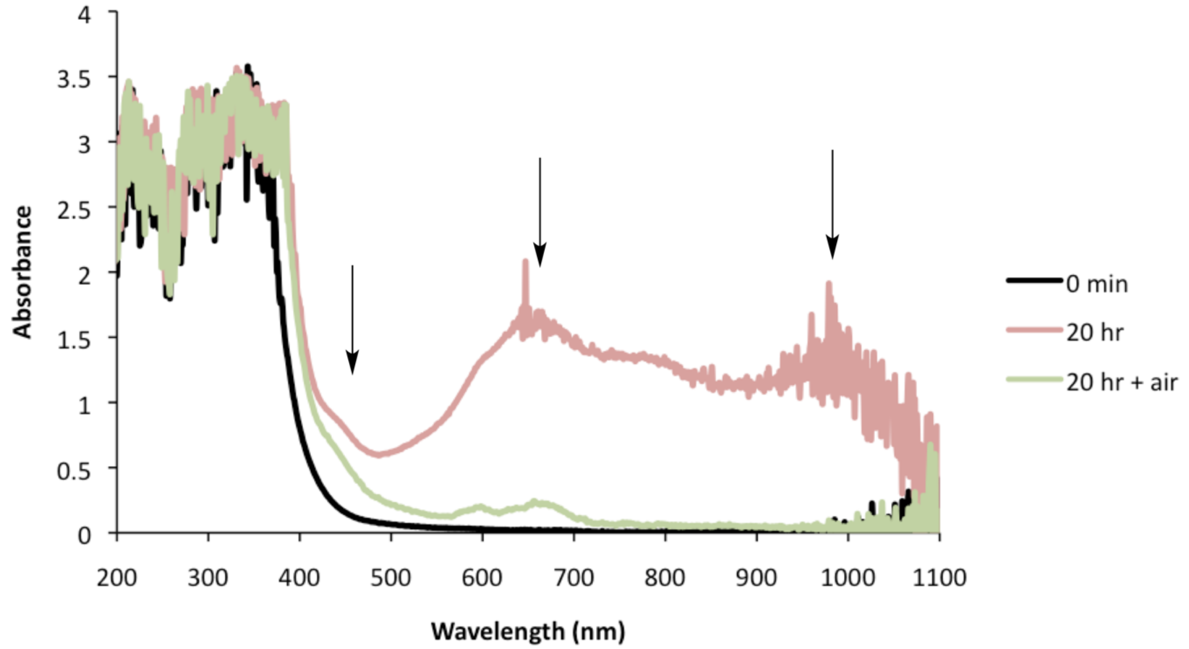

Supplementary Figure 14. Effect of oxygenation on the absorbance spectrum of continuous photolysis of a solution of toluene, 0.4 mol% TBADT, and 0.4 mol% COPC, absorbance spectrum

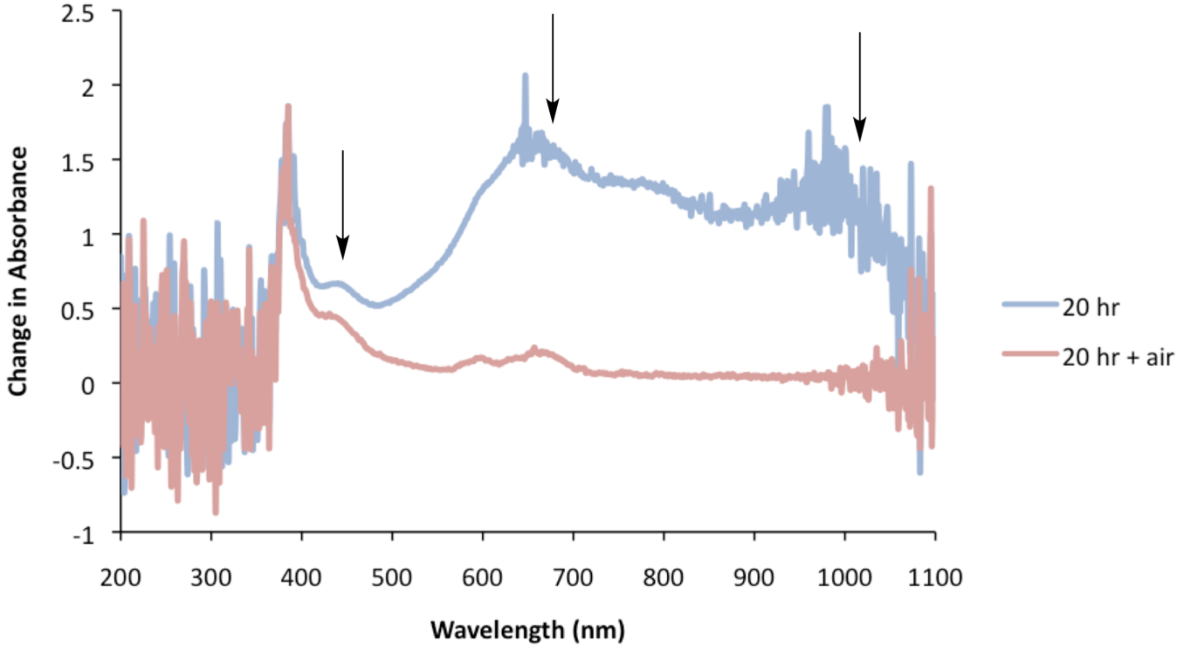

Supplementary Figure 15. Effect of oxygenation on the absorbance spectrum of continuous photolysis of a solution of toluene, 0.4 mol% TBADT, and 0.4 mol% COPC, difference absorbance spectrum

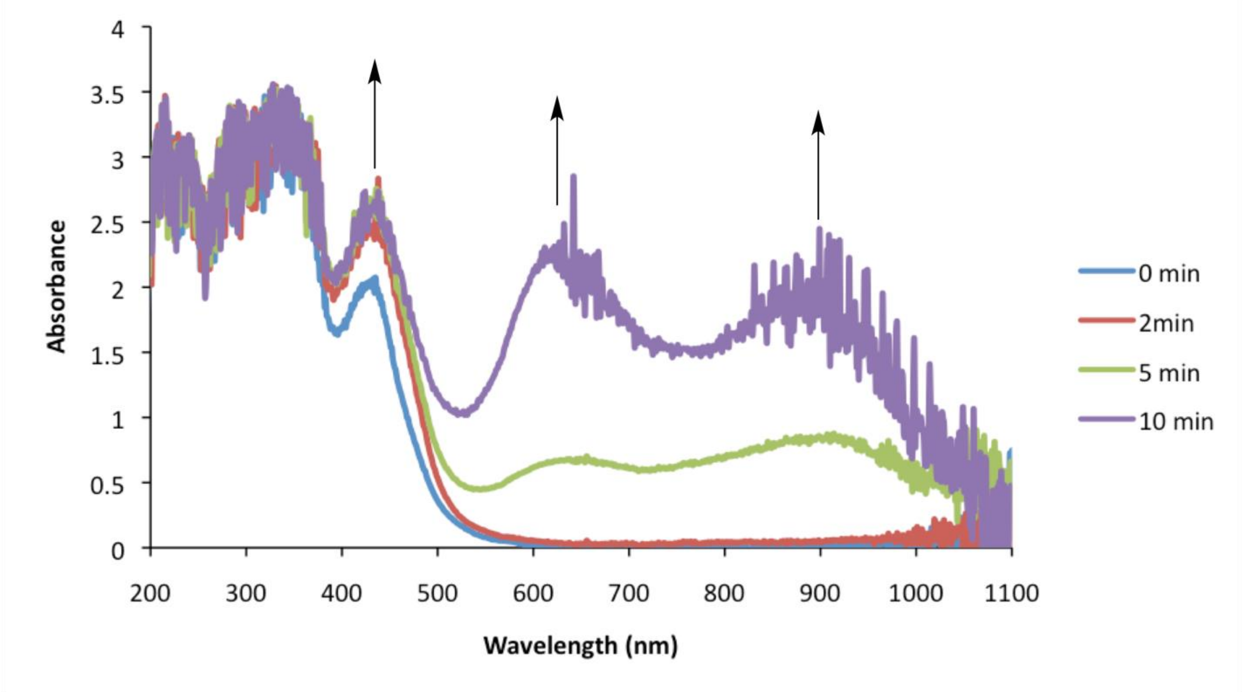

Supplementary Figure 16. Absorbance spectrum of continuous photolysis of a solution of cyclooctane, 0.4 mol% TBADT, and 0.4 mol% COBF for early time points

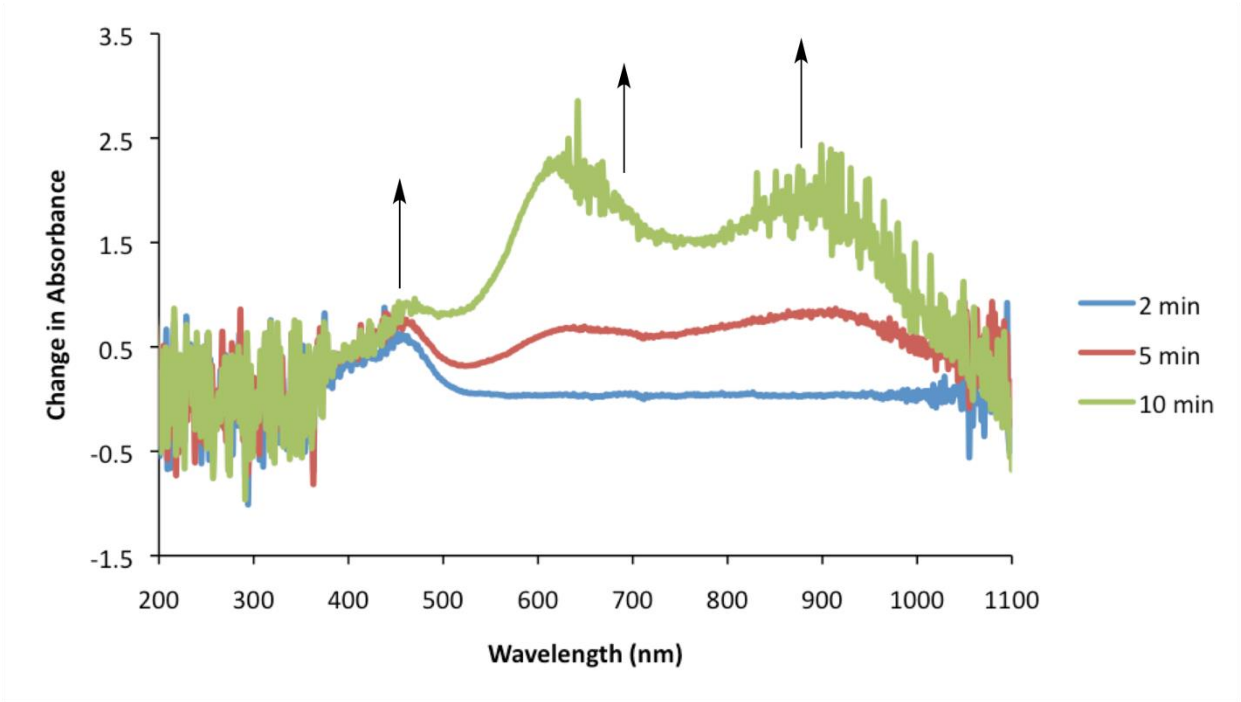

Supplementary Figure 17. Difference absorbance spectrum of continuous photolysis of a solution of cyclooctane, 0.4 mol% TBADT, and 0.4 mol% COBF for early time points

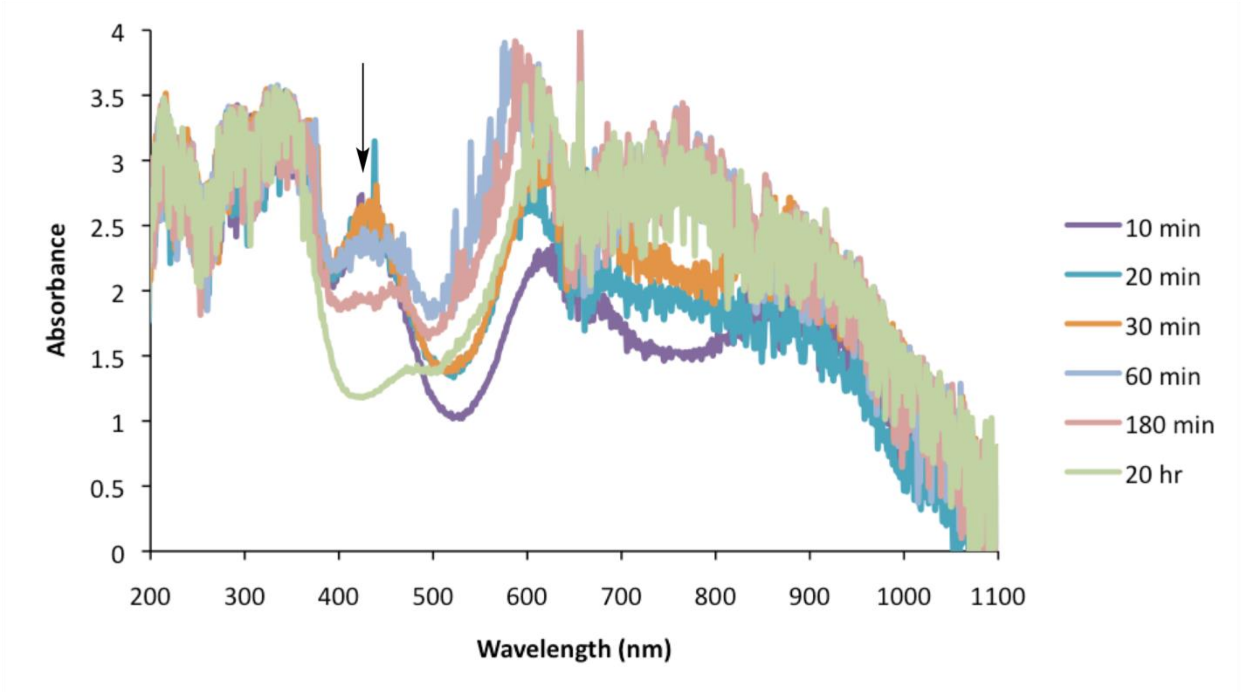

Supplementary Figure 18. Absorbance spectrum of continuous photolysis of a solution of cyclooctane, 0.4 mol% TBADT, and 0.4 mol% COBF for late time points

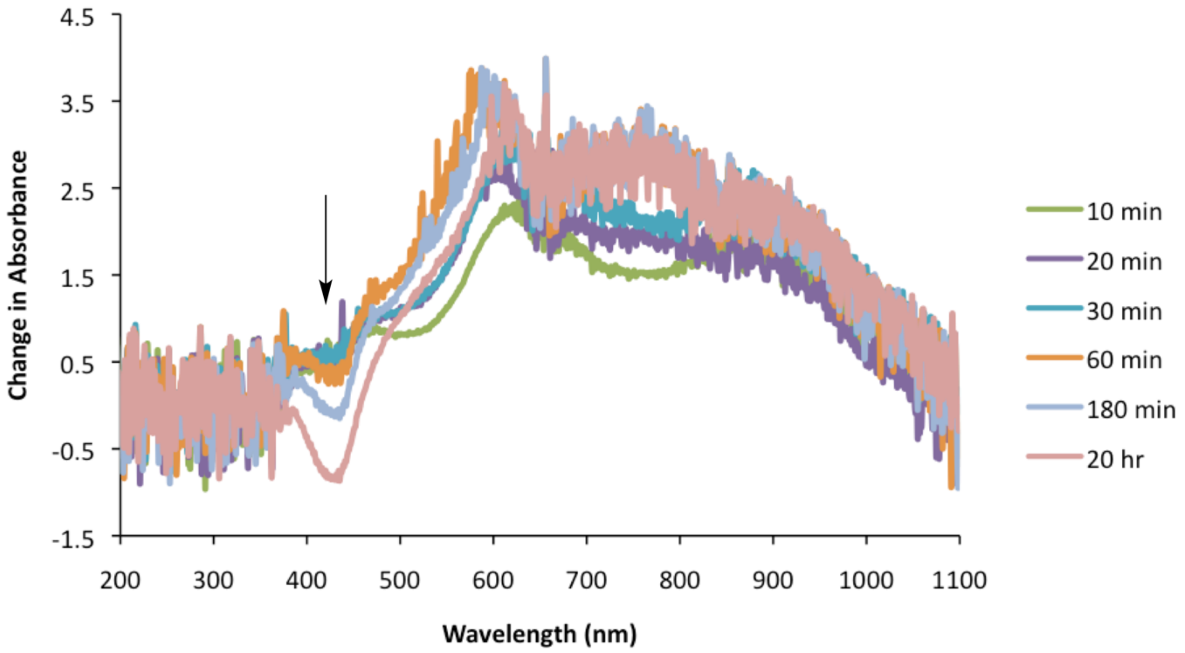

Supplementary Figure 19. Difference absorbance spectrum of continuous photolysis of a solution of cyclooctane, 0.4 mol% TBADT, and 0.4 mol% COBF for early time points

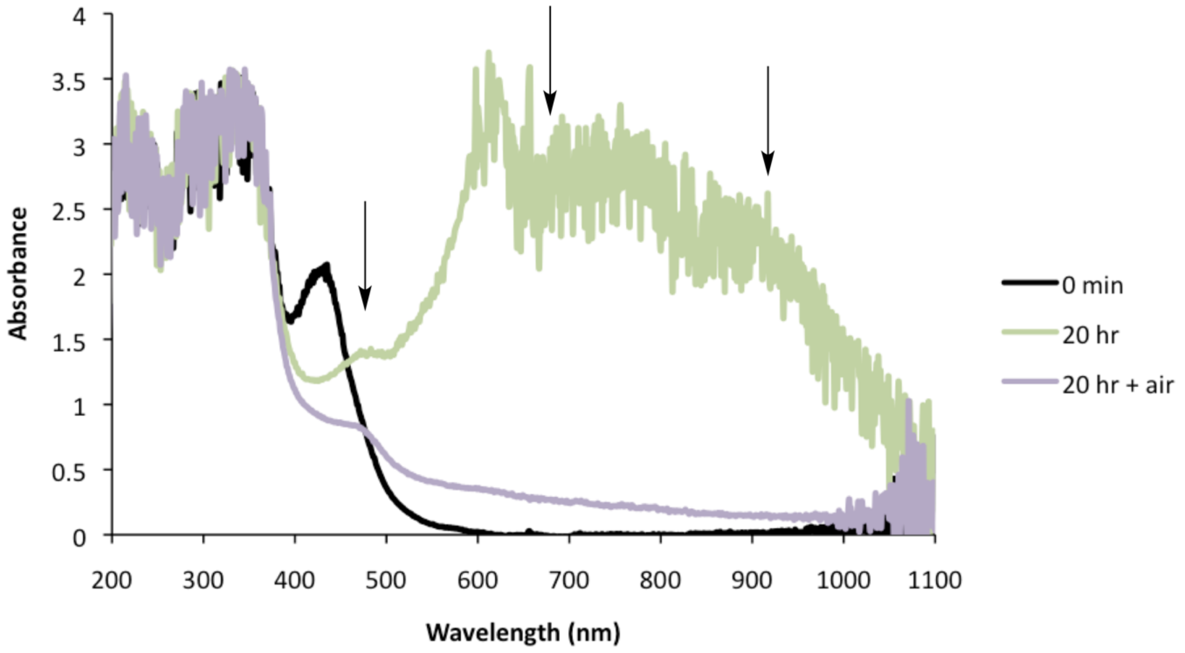

Supplementary Figure 20. Effect of oxygenation on the absorbance spectrum of continuous photolysis of a solution of cyclooctane, 0.4 mol% TBADT, and 0.4 mol% COBF, absorbance spectrum

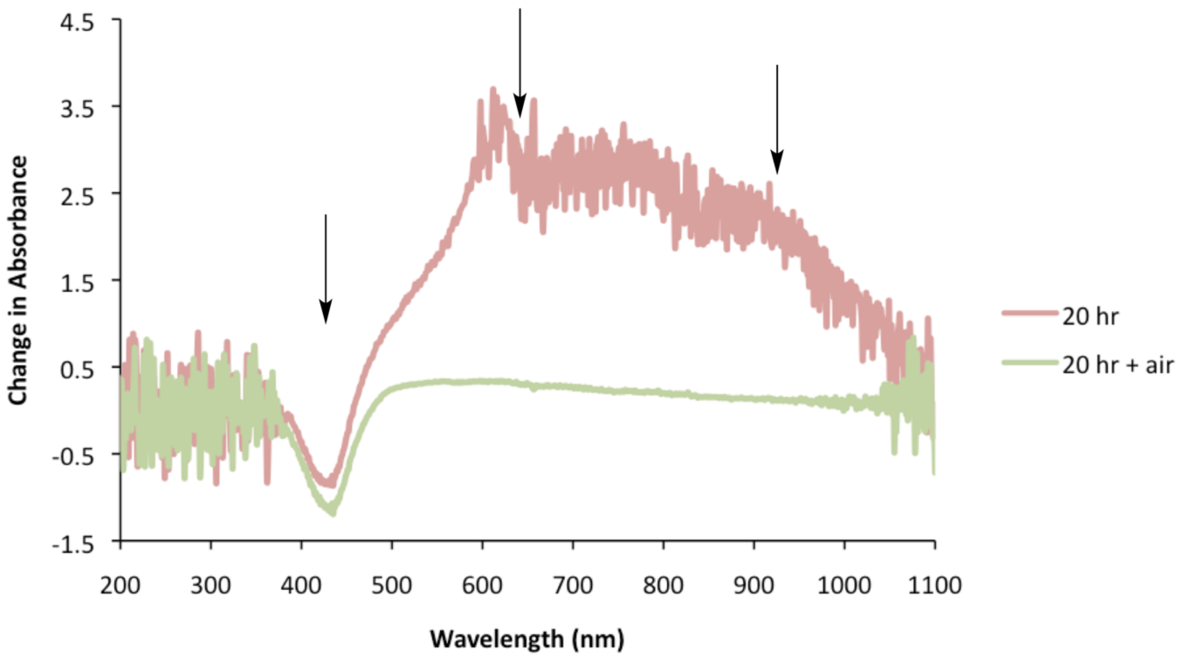

Supplementary Figure 21. Effect of oxygenation on the absorbance spectrum of continuous photolysis of a solution of cyclooctane, 0.4 mol% TBADT, and 0.4 mol% COBF, absorbance spectrum

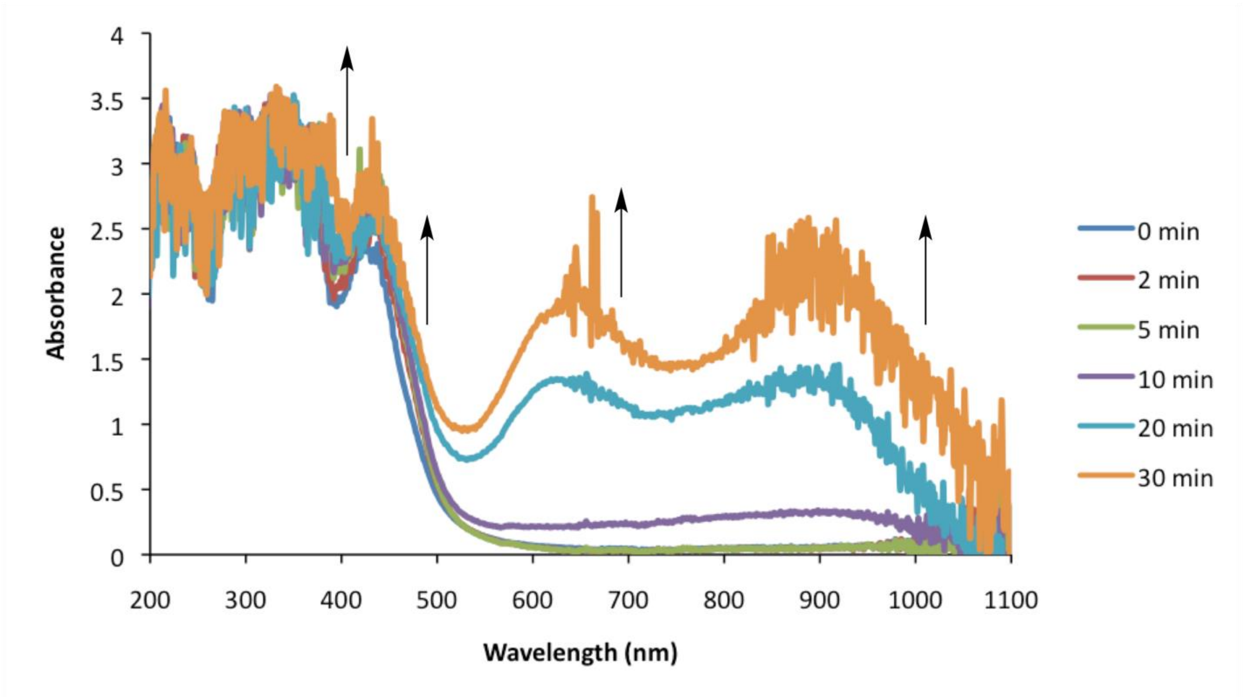

Supplementary Figure 22. Absorbance spectrum of continuous photolysis of a solution of toluene, 0.4 mol% TBADT, and 0.4 mol% COBF at early time points

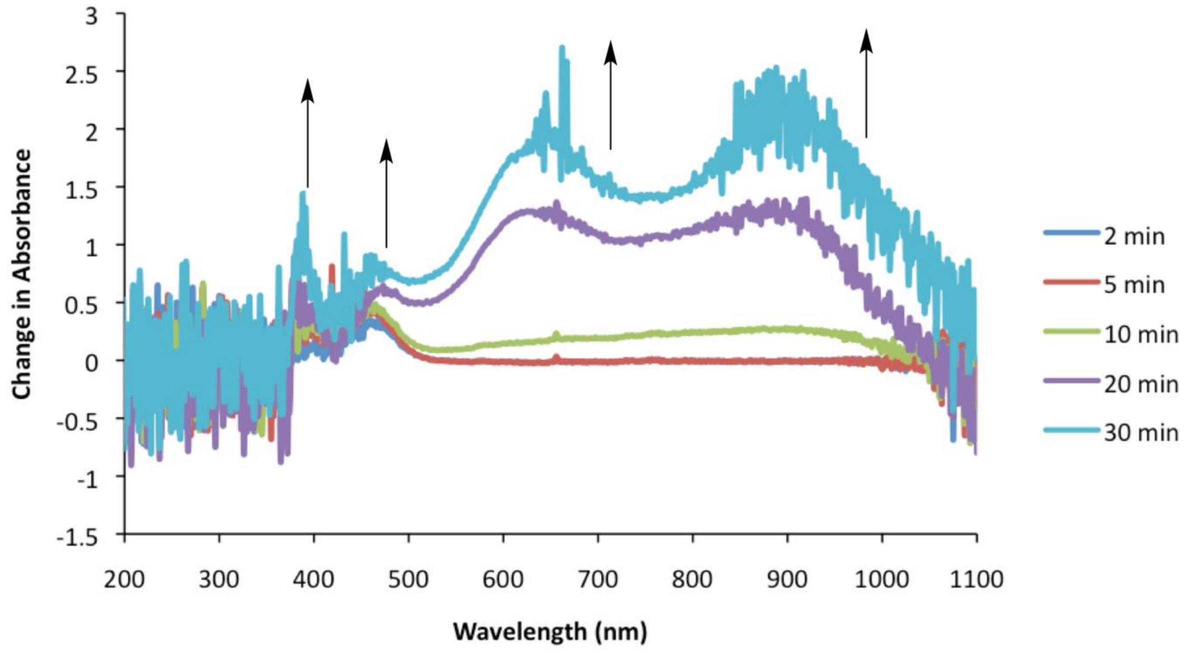

Supplementary Figure 23. Difference absorbance spectrum of continuous photolysis of a solution of toluene, 0.4 mol% TBADT, and 0.4 mol% COBF at early time points

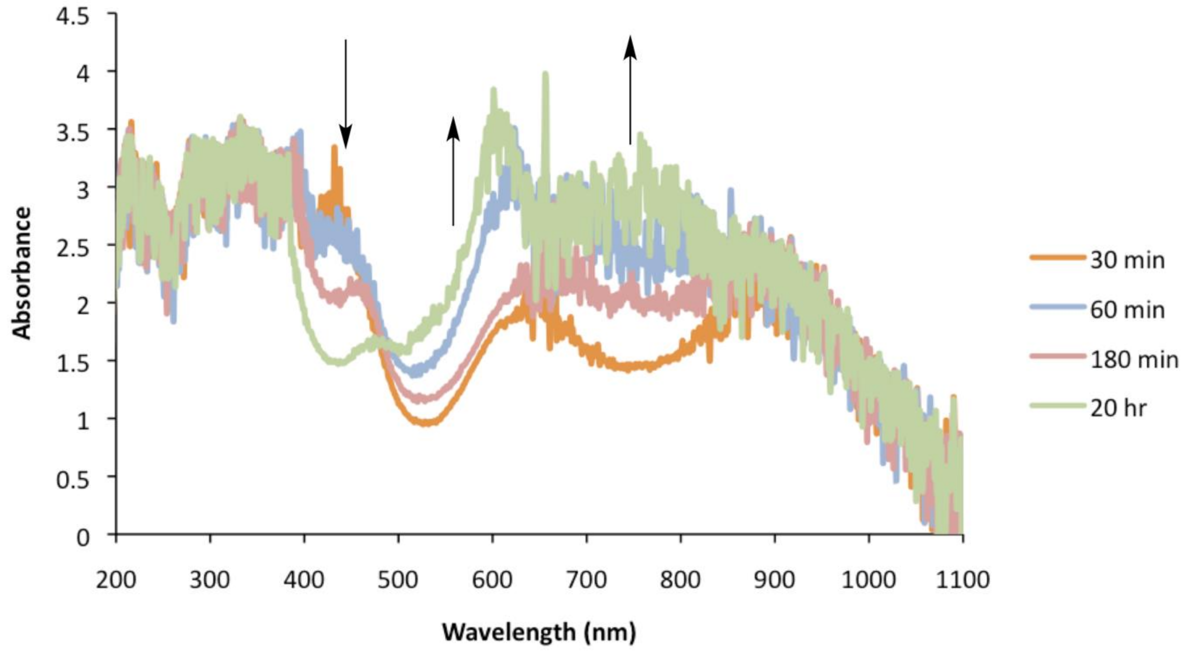

Supplementary Figure 24. Absorbance spectrum of continuous photolysis of a solution of toluene, 0.4 mol% TBADT, and 0.4 mol% COBF at late time points

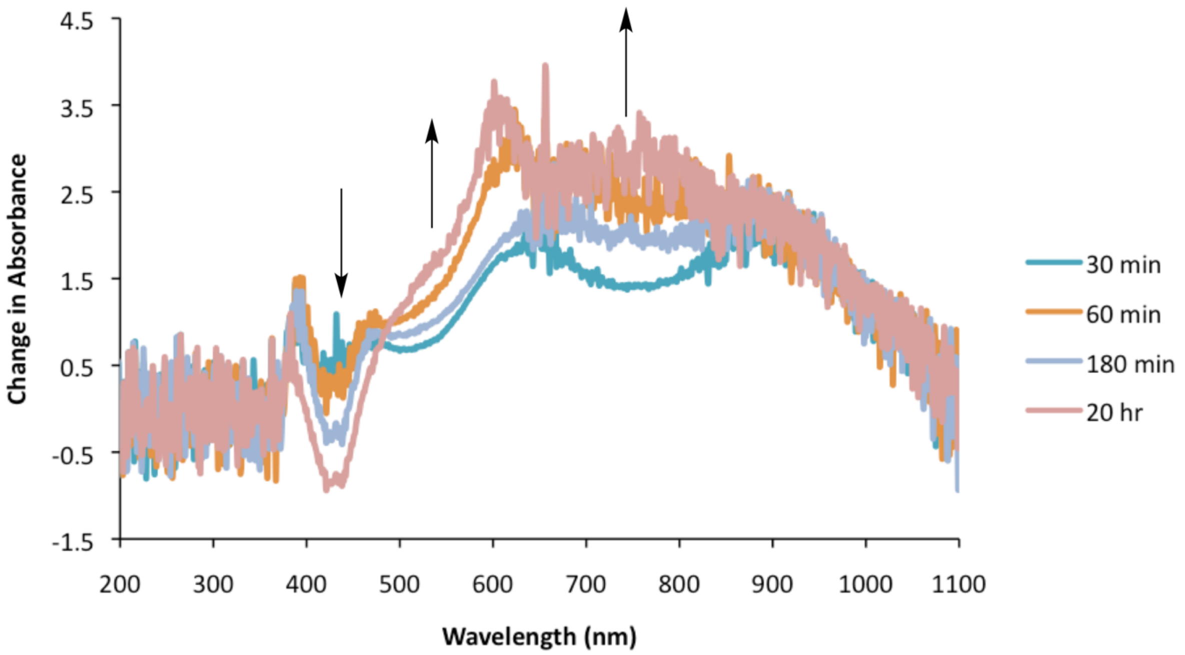

Supplementary Figure 25. Difference absorbance spectrum of continuous photolysis of a solution of toluene, 0.4 mol% TBADT, and 0.4 mol% COBF at late time points

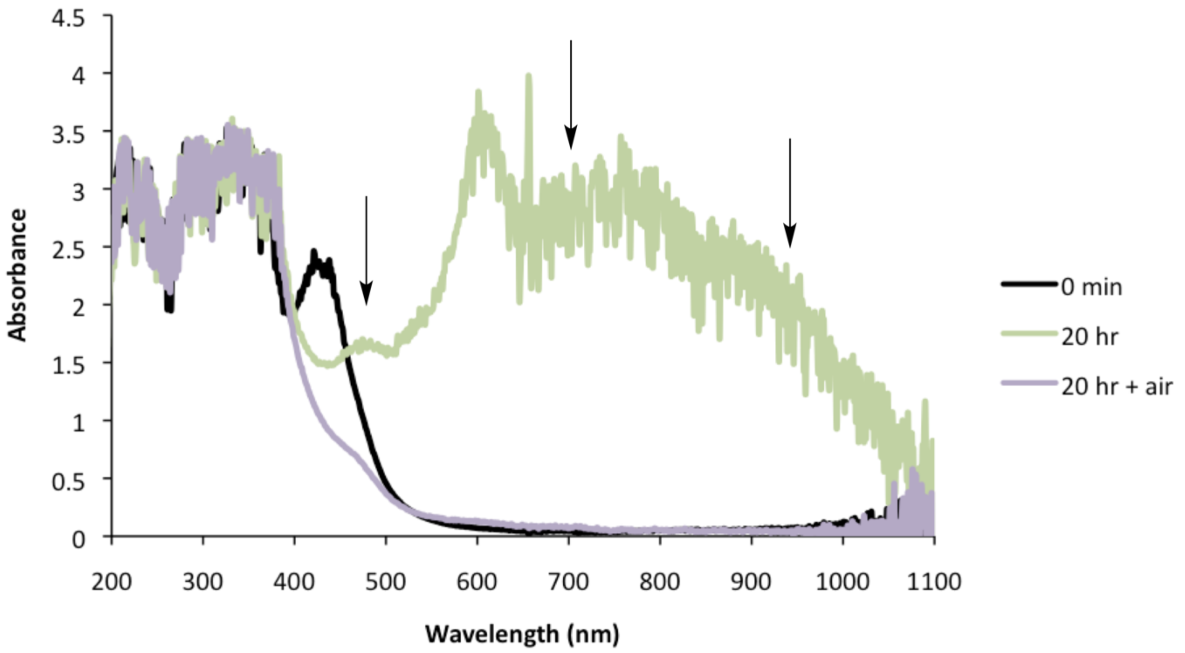

Supplementary Figure 26. Effect of oxygenation on the absorbance spectrum of continuous photolysis of a solution of toluene, 0.4 mol% TBADT, and 0.4 mol% COBF, absorbance spectrum

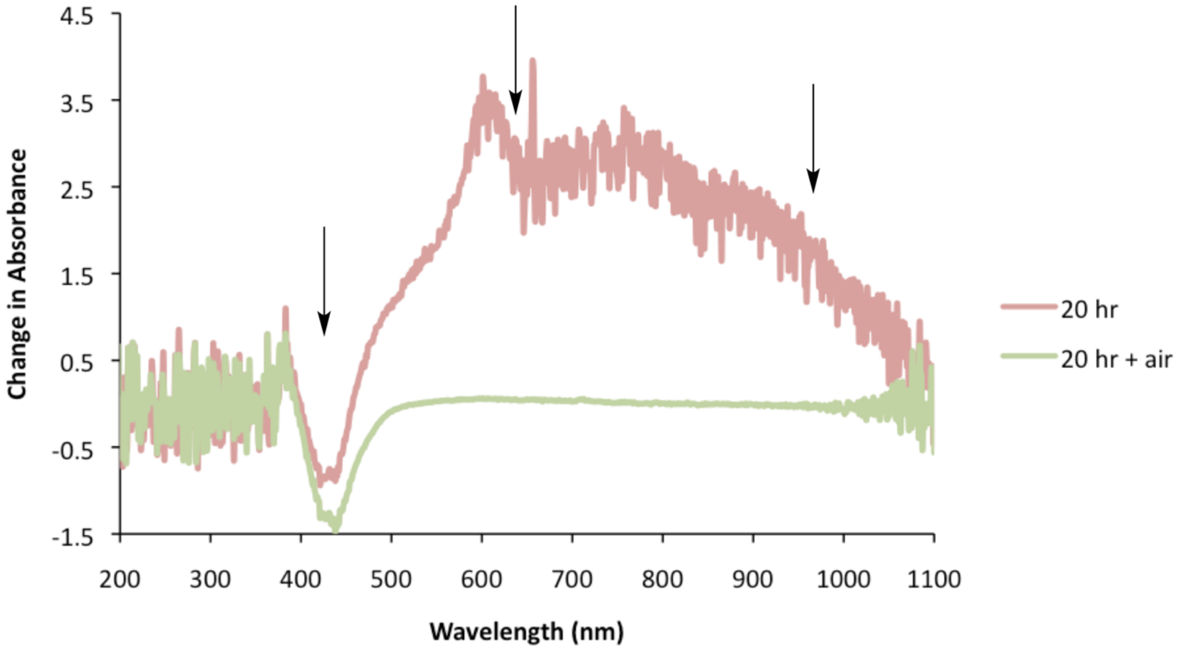

Supplementary Figure 27. Effect of oxygenation on the absorbance spectrum of continuous photolysis of a solution of toluene, 0.4 mol% TBADT, and 0.4 mol% COBF, difference absorbance spectrum

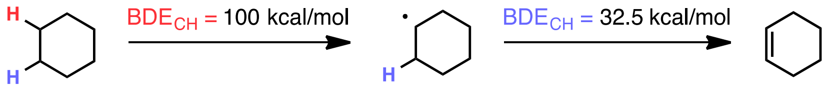

Supplementary Figure 28. The BDEs of stepwise dehydrogenated cyclohexane

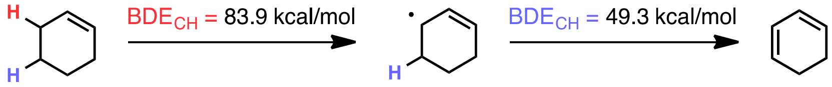

Supplementary Figure 29. The BDEs of stepwise dehydrogenated cyclohexene

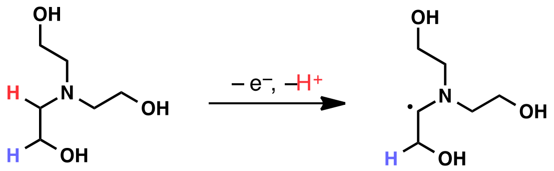

Supplementary Figure 30. The mechanism for the initial oxidation of triethanolamine

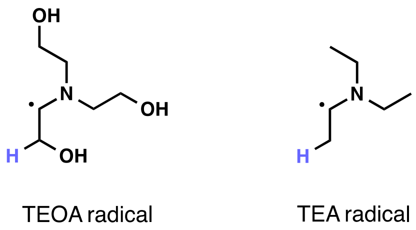

Supplementary Figure 31. The structures of the triethanolamine (TEOA) and triethylamine (TEA) α-amino radicals

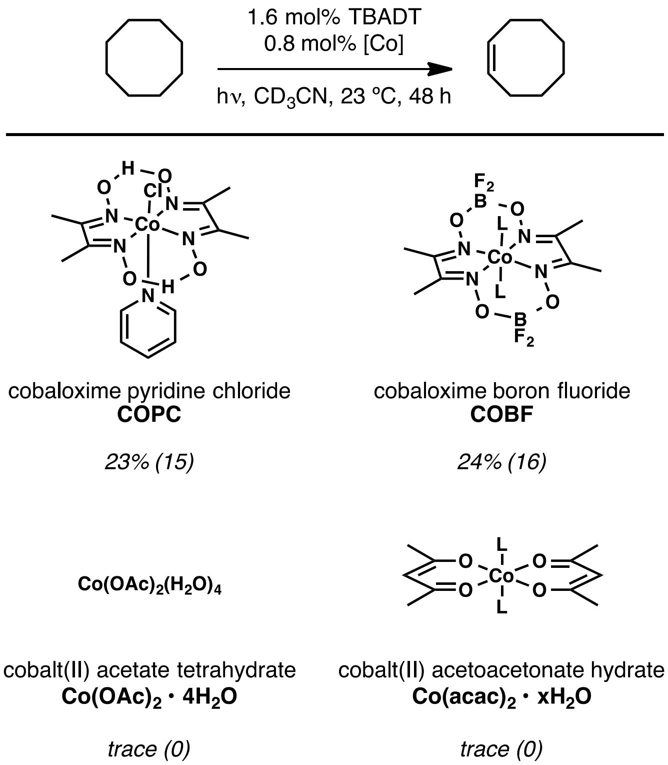

Supplementary Figure 32. cobaloximes are the only cobalt complexes that led to successful dehydrogenation. Results provided as yield% (cooperative TON).

Supplementary Table 1. Conversion of Starting Material as a Function of Conditions

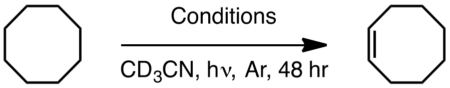

Entry	TBADT (mol%)	COPC (mol%)	Cyclooctene Yield (%)	Conversion (%)
1	0.4	0	trace	36
2	0.4	0.4	19	36
3	0	0.4	0	0
4	0	0	0	0

Supplementary Table 2. Selected Electrochemical Data of Investigated Complexes

Complex	Couple	E° (vs SCE in CH_3CN)	Reference
$W_{10}O_{32}^{4-}$	$W_{10}O_{32}^{4-}$ /W10O325−	-0.97	13
	$W_{10}O_{32}^{4-}$ /W10O325−	-1.53	13
COPC	Co(III)/Co(II)	-0.68	14
	Co(II)/Co(I)	-1.13	14
COBF	Co(III)/Co(II)	0.2	14
	Co(II)/Co(I)	-0.55	14
$Co(acac)_2$	Co(III)/Co(II)	-0.9	15
$Co(OAc)_2$	Co(III)/Co(II)	1.5	16

Supplementary Table 3. Additional exploration of alkane dehydrogenation conditions

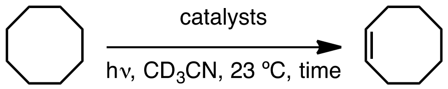

Entry	TBADT (mol%)	COPC (Mol%)	24 h Yield (%)	48 h Yield (%)
1	1.5	0	< 1	<1
2	1.5	0.75	14	23
3	1.5	1.5	14	20
4	3	0.75	12	25
5	0.75	0.75	11	19
6	0.4	0.75	8	11
8	0.4	0.4	12	19

Supplementary Table 4. Development of Alcohol Dehydrogenation Through Cooperative Catalysis

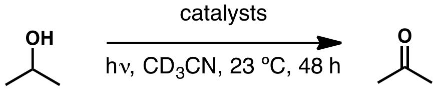

Entry	TBADT (mol%)	COPC (mol%)	Irradiation	Yield	Cooperative TON
1	2.0	0	yes	4	n/a
2	2.0	0	no	none	n/a
3	0	1.0	yes	none	n/a
4	0	1.0	no	none	n/a
5	2.0	1.0	yes	63	32
6	2.0	1.0	no	none	n/a
7	0.5	1.0	yes	17	17
8*	2.0	0	yes	11	n/a

Supplementary Discussion

On Mass Balance and Side Reactions

Reinvestigation of the optimized conditions and previous reactions reveals a consistent loss of mass balance across all dehydrogenation reactions that have been irradiated containing TBADT. A cursory analysis of the conversion of these representative reactions is provided in Supplementary Table 1.

These experiments show that the dehydrogenation substrate is consumed regardless of whether alkene is formed or not. This trend holds true for other substrates investigated, including cyclohexene and cyclopentane. Rigorous determination of the resulting products from this side reaction has proven challenging; however, some peaks in the ^{1}H NMR spectrum (in addition to mass spectrometry data) suggest that some of this may result from the production of the imine product through a sequence shown in Supplementary Fig. 1.

Indeed, this kind of reaction has been observed by Hill and coworkers upon extended photolysis of decatungstate solutions in acetonitrile.[1] Importantly, comparison of the TBADT/cobaloxime reactions with the TBADT/Pt^{o} case (where dimer is unambiguously produced) showed that cyclooctyl dimer, if formed, is in such small quantities that it cannot be detected via ^{1}H NMR.

Regardless of the exact nature of this side process, it is prudent to highlight several key points:

1) The formation of olefin using TBADT is **only** catalytic in the presence of a hydrogen evolution cocatalyst (either noble metal or cobaloxime)

2) The consumption of starting material occurs without respect to whether olefin is produced (catalytically) or not, even in reactions containing no hydrogen evolution catalyst

3) Dimer is not formed in detectable quantities under cobaloxime cocatalysis. Additionally, hydrogen production corresponds 1:1 with alkene formation, suggesting that it is the only relevant dehydrogenative process.

Taking all these points together, it seems likely that this unproductive conversion is due to competitive side-reaction(s) mediated by TBADT. This factor, while complicating, does not invalidate the mechanistic hypothesis of this reaction.

Electrochemical Data

All electrochemical data are from previous report1[,2,3,4] and were measured in acetonitrile. If the potential was not measured against the standard calomel electrode (SCE), it was converted to its value vs SCE as described by Addison.[5]

As can be seen from the electrochemical data (Supplementary Table 2), even the singly-reduced decatungstate ($W_{10}O_{32}^{5-}$) is sufficiently reducing to produce the Co(II) by reduction of the Co(III) species of all complexes tested. Additionally, the doubly-reduced decatungstate ($W_{10}O_{32}^{6-}$) is reducing enough to produce Co(I) species from both COPC and COBF. The reduction potentials of cobalt acetylacetonate and cobalt acetate for the Co(II)/Co(I) couple in acetonitrile were not able to be found by these authors, however it is believed that they are non-trivial.

Singly-reduced decatungstate is known to disproportionate into both the ground-state and double-reduced species, making the electrochemical possibilities quite numerous for these reactions. In part due to this complexity, UV-Vis was chosen as a more insightful method of characterizing the reaction, especially as it can be monitored under catalytically-relevant conditions.

UV-Vis Continuous Photolysis Experiments

One of the preeminent methods for the study of both TBADT and cobaloxime reactions, individually, under catalytically-relevant conditions has been UV-Vis spectroscopy. We sought to gain more insight into the combined catalyst system using this method.

The first continuous photolysis experiment that was pursued is the irradiation of cyclooctane in the presence of 0.4 mol% TBADT. As can be seen from the absorbance spectrum (Supplementary Fig. 2), an immediate growth of a broad absorbance characteristic of reduced TBADT (with absorbtion maximum at 630 nm, consistent with

the observations of Tanielian[6]) is observed even after 2 minutes of irradiation. The growth of this feature continues over the course of the photolysis and is joined by a similarly diagnostic peak at 780 nm after 60 minutes of irradiation.

Examination of the difference absorbance spectrum of the reaction (Supplementary Fig. 3) accentuates the growth of the aformentioned absorbances while also revealing a bleaching of the reaction’s absorbance at ~350 nm. Again, this behaviour is consistent with the photoreduction of TBADT and provides a valuable baseline for the reaction.

Following 150 minutes of irradiation, the reaction was exposed to oxygen which led to the absorbance spectrum returning to its pre-irradiation values (figs 4 and 5). This experiment suggests that the decatungstate is not meaningfully consumed during the course of this non-catalytic dehydrogenation.

NMR analysis of the reaction post-aeration showed a detectable, albeit small, amount of cyclooctene, a result consistent with our earlier observations.

A logical next step for the analysis of the reaction is the photolysis of a solution containing cyclooctane and COPC. As can be seen in both the absorbance and difference spectra (supplementary figs 6 and 7), there is essentially no change to the reaction mixture following 120 minutes of irradiation, an assertion that is backed up by the absense of any cyclooctene product in an NMR following irradiation.

Having established that TBADT is the main photoactive component of the reaction mixture (at least at early time points), a reaction reflective of the optimal conditions, cyclooctane, 0.4 mol% TBADT, and 0.4 mol% COPC, was subjected to continous photolysis. Interestingly, the appearance of absorbances corresponding to reduced decatungstate is delayed until ~ 30 minutes of irradiation, with hitherto unobserved peaks at 430, 590, and 655 nm growing in during the preceding period (Supplementary Figs. 8 and 9). The peak at 430 nm is consistent with the reduction of the Co(III) center in COPC to Co(II),[7] a reaction that should readily occur according to the relative reduction potentials of COPC and reduced decatungstate (–0.68V and –0.97V, respectively; both vs SCE). The lower energy absorbances at 590 and 655 nm do not seem to have as direct precedent as that at 430 nm, however similarly-shaped features in the same wavelength range (550-700 nm) have been assigned to Co(I) species for both COPC and the related COBF in prior reports.[8] The electrochemistry for the reduction of the Co(II) species to Co(I) by the singularly-reduced decatungstate, however, is significantly less compelling (–1.13V and –0.97, both vs SCE). It is possible that the reduced decatungstate could disproportionate into the ground state and doubly-reduced species, a known reaction whose product is sufficiently reducing (–1.53V vs SCE) to produce the Co(I) species, however several other features of this reaction further discourage this thinking:

1) no absorbances corresponding to reduced decatungstate are observed during the growth of these features, suggesting that the concentration of this species is quite low and, as a result, bimolecular disproportionation seems unlikely

2) the appearance of the "Co(I)" feature is concurrent with the growth of the Co(II) absorbance. In combination with the decatungstate concentration argument above, we do not expect it to be competitive to reduce Co(II) in the presence of a large excess of Co(III)

3) These same features are observed in the uncatalytic toluene reaction (see below)

4) Aeration of the reaction does not completely bleach this feature (Co(I) is known to be air sensitive).

Taken together, we hesitate to assign this absorbance as Co(I). Interestingly, recent reports by both Guldi[9] and Peters[10] have shown the formation of dimeric cobaloxime species to be of potential relevance to their hydrogen evolution reactions. These associations seem to exhibit equilibrium behaviour and their formation from both same and mixed oxidation state monomers (Co(II)/Co(II) and Co(II)/Co(III)) provides many possibilities for them to occur in these reactions.

Regardless of the identity of the peaks at 590 and 655 nm, the broad absorbance assignable to reduced TBADT (in analogy to Supplementary figs. 2 and 3) becomes a dominant feature of the reaction's spectrum following 30 minutes of irradiation. While this absorbance somewhat obscures any changes that might occur to the aforementioned peaks, the Co(II) absorbance at 430 nm remains well resolved throughout the reaction. Interestingly, the intensity of this peak plateaus after 30 minutes and only marginally decreases after 20 hours of irradiation. One possible explanation for this observation is Co(II) being the resting state for the COPC-catalyzed reaction, a not-unreasonable assertion as Co(II) is known to be the active oxidation state for cobalt-mediated hydrogen-atom-transfer processes. The small decrease in intensity following prolonged irradiation could be due to decomposition, however this is by no means definite. Importantly, the presence of even small amounts of Co(II) is capable of suppressing radical polymerization and disproportionation, an observation that is consistent with the results of these reactions.

Exposure of the reaction to air after 20 hours of irradiation leads to the immediate disappearance of features assignable to reduced decatungstate and the Co(II) cobaloxime, two species that are expected to be exceptionally air sensitive (Supplementary Figs. 10 and 11). Conversely, the peaks at 590 and 655 nm remain at intensities largely unchanged from those before the appearance of reduced decatungstate features, again inconsistent with it corresponding to Co(I). NMR assay of the aerated reaction shows a moderate amount of cyclooctene, suggesting catalysis occurs under these conditions.

A final aspect of note is the appearance of a new peak at ~385 nm after 20 hours of irradiation as shown by the difference spectrum (Supplementary Fig. 9). This peak is not readily assignable on its own, however we tentatively propose that it might correspond

to an alkyl Co(III) species due to the formation of an analogous peak in the photolysis of a toluene reaction (*vide infra*)

The photolysis of an analogous solution of toluene, 0.4 mol% TBADT and 0.4 mol% COPC presents quite similar features in the early stages of the reaction (2-10 minutes, Supplementary Figs. 11 and 12) to that of the cyclooctane reaction. Interestingly, after 20 minutes of irradiation a peak at ~ 385 nm begins to become prominent in the difference spectrum (Supplementary Fig. 13), only growing in intensity as the photolysis continues. Most critically, this feature is not significantly diminished following aeration of the reaction solution, a characteristic that would be expected from a Co(III) species (Supplementary Figs. 14 and 15). Additionally, alkyl Co(III) species are known to absorb in this wavelength range more strongly than either Co(I) or Co(II) cobaloximes,[11] giving some support to this proposal.

While it is tempting to point to catalyst deactivation through the formation of alkyl-cobaloximes as the sole cause of the failure of toluene dimerization in this reaction and, by extension, casting doubt on the relevance of the cHAT proposal, it is prudent to draw one's attention to more features of the reaction:

1) cHAT is not mutually exclusive with radical recombination between Co(II) and carbon-centered radicals. Indeed, if the peak at 385 nm is to be assigned as alkyl-Co(III), it can be observed that a small amount of this is formed after 20 hours in the cyclooctane photolysis. However, it is essential to note that no other possibilities exist (ie HAT from a center adjacent to the radical as proposed for the cyclooctyl case) in the case of toluene. Therefore, any interaction between Co(II) and the benzyl radical should result in the formation of benzyl cobaloxime.

2) If the cobaloxime is serving as only an electrocatalyst this reaction should still be competent for TBADT turnover. Indeed, the aeration of the reaction mixture shows that some air-sensitive Co(II) species are still present after 20 hours of irradiation (through the disappearance of the 430 nm absorbance). As this is not observed (ie only stoichiometric formation of bibenzyl), it is doubtful that the cobaloxime is functioning as a simple hydrogen-evolving electrocatalyst for reduced decatungstate.

3) Benzyl cobaloximes are known to be readily photolyzed under UV-irradiation, regenerating the Co(II) species and benzyl radical.[12,13]

The identity of the peak at 385 nm aside, it can be seen that the absorbance of the features relating to reduced decatungstate in the toluene photolysis of COPC does not reach the same intensity as that of the cyclooctane reaction (Supplementary Fig. 8), despite having a similar decatungstate concentration. There is not an obvious explanation for this observation.

Another set of experiments that appeared to be meritorious to our study of this reaction is the photolysis of cyclooctane and toluene using the other successful cobalt cocatalyst, COBF. Irradiating a solution of cyclooctane, 0.4 mol% TBADT and 0.4 mol%

COBF led to a much more rapid growth of features assignable to the reduced decatungstate than observed in the COPC-mediated reaction, with significant intensity for these features after only 5 minutes of irradiation (Supplementary Figs. 16 and 17). Before this (2 and 5 minute time points), it can be seen that the absorbance assigned to Co(II) (430 nm) increases in intensity. As COBF is a Co(II) complex, the intensity of this peak should not increase compared to the time zero point. A likely cause of this observation is partial oxidation of the COBF prior to the reaction, as reaction preparation was carried out under ambient atmosphere (it is known that COBF is *resistant but not immune* to aerial oxidation compared to other Co(II) cobaloximes). An important contrast to the COPC reactions is the complete absense of low-energy peaks at ~590 and 650 nm during the early stages of this reaction. As reducing COBF to Co(I) should be significantly more facile than reducing COPC, this provides further evidence against these absorbances corresponding to the COPC Co(I) species.

Following the reduction of this putative Co(III) population (5 – 10 minutes, Supplementary Figs. 16 and 17), the reaction adopts a feature approximating an isosbestic point at ~450 nm, a known feature for COBF reactions cycling between Co(II) and Co(I) oxidation states (Supplementary Figs. 18 and 19). Indeed, a gradual bleaching of the 430 nm Co(II) absorbance is accompanied by a growth of a lower-energy feature that overlaps with the reduced decatungstate peaks. This low energy absorbance is consistent with the observations of Peters[14] and Norton[7] for the Co(I) oxidation state which, together with the aforementioned pseudo-isosbestic point, is suggestive of its accumulation over the course of the reaction.

There are two possible (and not mutually exclusive) rationales for this observation. The first, again, has its roots in the electrochemistry of the system: even the singly-reduced decatungstate (W^{4-}/W^{5-} -0.97 V vs SCE) should be sufficiently reducing to transfer an electron to COBF (Co^{II}/Co^{I} -0.55 V vs SCE). While certainly possible, one further observation that casts some doubt on this being solely responsible for hydrogen evolution is the accumulation of a large amount of reduced tungstate before the concentration of Co(II) begins to decrease. It was observed in the COPC reactions that an electron transfer process (i.e. reduced tungstate to Co(III)) can occur quite rapidly and prevent the accumulation of reduced tungstate (see time points 2 through 20 minutes of Supplementary Figs. 8 and 9 for an example), a similar trend as seen for time points 2 and 5 minutes of this system. Of course, it is possible that the direct reduction of Co(II) by reduced decatungstate is a competitive (potentially unproductive) side reaction that proceeds during the course of the reaction, slowly depleting the amount of Co(II).

A second possibility comes from consideration of the proposed cHAT catalytic cycle, namely that the putative “hydridocobaloxime” is an essential component of the cobalt cycle. Recent reports by Norton[7] and Peters[14] have provided compelling evidence for the identity of this species not being the cobalt-bound Co(III) hydride but, rather, a Co(I) center with a protonated ligand oxygen. Indeed, subjecting COBF in acetonitrile to high pressure (70 atm) of H_2 leads to the gradual conversion of Co(II) to Co(I) as observed by UV-Vis, with spectral features (absorbance changes and isosbestic point) very

similar to the current system. Taking this together, it could be possible that this change in absorbance corresponds to the formation of the “hydridocobaloxime” produced through hydrogen atom transfer from the alkyl radical intermediate.

Prolonged irradiation (180 minutes and longer) leads to the discontinuation of this pseudo-isosbestic point with continued depletion of the Co(II) spectral feature. Following 20 hours of irradiation, the reaction was exposed to ambient atmosphere and the observed spectrum shows a marked decrease in the amount of Co(II) when compared even to the 0 time point (Supplementary Figs. 20 and 21). One interpretation of this data is that the cobaloxime is degraded during the course of the reaction, an observation that would not be entirely unprecedented in hydrogen evolution reactions catalyzed by COBF.

Analysis of the aerated reaction mixture by NMR revealed a significant accumulation of cyclooctene, a result consistent with the catalytic relevancy of these conditions.

Performing the toluene photolysis with 0.4 mol% TBADT and 0.4 mol% COBF (Supplementary Figs. 22 and 23) leads to an outcome not completely unexpected based on the results of the COPC toluene photolysis and the COBF cyclooctane reaction. To explain, a similar introductory period leads to an increase in the Co(II) absorbance with no detectable accumulation of reduced decatungstate. Once the 430 nm peak has reached its maximal value (~ 20 min, Supplementary Figs. 22 and 23), the reduced decatungstate begins to appear in the reaction, again consistent with a reduction of Co(III) impurity prior to the beginning of the catalytic reaction (Supplementary Figs. 24 and 25).

Much like with the cyclooctane case, a pseudo isosbestic point is established following this introductory phase that is consistent with a Co(II)/Co(I) reaction mixture. Depletion of the Co(II) absorbance is again accompanied with growth in the lower energy region of the spectrum that could correspond to the production of Co(I). However, an important contrast with the COBF cyclooctane photolysis is the growth of a high energy band at 385 nm that is analogous to that observed in the COPC toluene photolysis (Supplementary Figs. 12 and 13). Again, this might tentatively be assigned as the production of a Co(III) benzyl species as discussed for the COPC toluene reaction. Exposure to air again leads to similar behaviour to the cyclooctane case (Supplementary Figs. 26 and 27)

Discussion of Bond Strengths

A striking feature of the disclosed dual catalytic system is its result when cyclohexane is the substrate, namely that the main product is cyclohexene and not benzene. Analysis of the first dehydrogenation yields the expected result of the second hydrogen atom’s BDE[15,16] being much lower than the first (Supplementary Fig. 28)

Interestingly, the compared to this first case, the second dehydrogenation reaction (to make cyclohexadiene) proceeds via initial abstraction of a lower energy hydrogen atom

to make a stabilized allyl radical whose second BDE[15] is significantly **higher** than in the first dehydrogenation (Supplementary Fig 29)!

This result can be understood through ground state energy-lowering of the allyl radical intermediate compared to a 2º alkyl radical of the previous case. As the “cobalt hydride” strength of COBF has been calculated to be 50.5 kcal/mol,[7] the thermodynamics of this second HAT, while still favourable, are not nearly as compelling as observed for the initial dehydrogenation event. The potential for reversible HAT (as observed in catalytic chain transfer) would be consistent with both the predominance of cyclohexene in the product distribution and the low overall efficiency of the process.

A potential implication of the importance of C–H BDEs to cobaloxime hydrogen evolution catalysis is found in the photocatalytic hydrogen evolution literature, mainly in the predominance of tri(ethanol)amine (TEOA) as the sacrificial reductant. Early studies by Gratzel[17] on the photosensitized oxidation of TEOA established that an intermediate product in this reaction is the α-amino radical. TEOA then must lose one more hydrogen atom equivalent to become fully oxidized (supplementary Fig. 30).

In the course of studying eosin-sensitized hydrogen evolution using COPC, Eisenberg and coworkers[8] found that the substitution of TEOA with triethylamine (TEA) led to a dramatic decrease (greater than 20 times, supplementary Fig 31) in the efficiency of the reaction. Furthermore, the attempted substitution of alkylamines with inorganic reductants whose potentials are compatible with the sensitizer (NaI, Na_2S, $Na_2S_2O_4$) led to no hydrogen production. Taking this all together, we propose that a possible cause of the singular success of alkylamines in these reactions is due to the ability of their α-amino radicals to function as effective hydrogen atom donors. Indeed, first approximation suggests that the TEOA radical to be a better hydrogen atom donor than the triethylamine equivalent from the additional, labilizing effect of having an α-oxygen functionality.

More work is required to substantiate this assertion; however, it is entirely consistent with the dual function of cobaloximes as both HAT and hydrogen evolution catalysts.

Mechanistic Summary

While the H_2 evolution step remains obscure, certain aspects of the proposed reaction are quite consistent with the cHAT proposal.

• In both the COPC and COBF reactions, it is shown that the concentration of Co(II) in the reaction mixture is significant for the majority of the reaction, even after 20 hours of irradiation. This population of active CCT catalyst should be able to suppress radical-radical coupling reactions through HAT chemistry, an assertion that is supported by the lack of dimer formation in the cyclooctane dehydrogenation and the 1:1 stoichiometry of hydrogen and alkene production. Indeed, even if the cobaloxime is regenerating the TBADT catalyst through canonical, electrocatalytic hydrogen evolution, a more-than-

adequate amount of free cobaloxime is maintained throughout the reaction to perform the second, rapid HAT that is the key feature of cHAT.

• The reduction of Co(III) precatalyst to form an active Co(II) species occurs during the early stages of the COPC-mediated reactions. This transformation is signaled by the growth of a Co(II) absorbance with no reduced decatungstate being detectable. Once this reduction is complete, reduced decatungstate begins to accumulate in the reaction mixture. This growth of reduced decatungstate features without a corresponding bleach of the Co(II) absorbance for the COPC cyclooctane reaction suggests that the further reduction of the Co(II) species to Co(I), as proposed in electrocatalytic hydrogen evolution schemes, is slow, if it occurs at all.

• It is possible that benzyl cobaloximes are formed during the course of the toluene photolysis reaction, however some Co(II) species remain in solution even after 20 hours of irradiation for both the COPC and COBF-mediated reactions. If the cobaloxime is serving as an electrocatalytic hydrogen evolution catalyst, it should still be able to regenerate the TBADT in the toluene case and a significant amount of bibenzyl should be observed; this is not the case. Additionally, it is known[12,13] that benzyl cobaloximes are readily homolyzed under UV-irradiation, making the formation of these species an unlikely cause of the non-catalytic behavior of the toluene reaction.

At the very least, the synergistic HAT proclivities of TBADT and cobaloximes lead to an idiosyncratic selectivity for alkenes over other radical disproportionation/polymerization products, the central tenant of this designed reaction platform.

Supplementary Methods

General Information

Commercial reagents were purified prior to use following the guidelines of Perrin and Armarego.[18] All solvents were purified by column according to the method of Grubbs.[19] Organic solutions were concentrated under reduced pressure on a Büchi rotary evaporator. Chromatographic purification of products was accomplished using force-flow chromatography on Aldrich silica gel according to the method of Still.[20] Thin-layer chromatography (TLC) was performed on Aldrich 250 μm silica gel plates. TLC visualization was performed by fluorescence quenching, $KMnO_4$ or iodine stain. All yields reported are averages of at least two experimental runs.

^{1}H spectra were recorded on a Bruker 500 (500 MHz) and are referenced relative to residual CD_3CN or $CDCl_3$ proton signals at δ 1.94 ppm and δ 7.26 ppm, respectively. Data for ^{1}H are reported as follows: chemical shift (δ ppm), multiplicity (s = singlet, d = doublet, t = triplet, q = quartet, m = multiplet, b = broad, ap = apparent), integration, coupling constant (Hz) and assignment. ^{13}C spectra were recorded on a Bruker 500 (126 MHz) and are referenced relative to CDCl at δ 77.23 ppm. Data for ^{13}C NMR are reported in terms of chemical shift and multiplicity where appropriate. High Resolution

Mass spectra were obtained from the Princeton University Mass Spectral Facility. Hydrogen gas production was confirmed via headspace gas analysis using a Hewlett-Packard 5980 GC with an agilent CP7538 column and a thermal conductivity detector with column head pressure of 12 psi, oven temperature of 70 °C, injection temperature of 200 °C, and a detector temperature of 250 °C. Hydrogen gas production was quantified through the use of a 5 cc gas syringe that was used to equilibrate the reaction vessel pressure with atmospheric pressure. UV-Vis measurements were acquired using an Agilent 8453 UV-Visible Spectroscopy System.

Reaction Set-up

In a typical experiment, a 26 W compact fluorescent light bulb (Exo-Terra Reptile 150) was chosen because of the combination of its intense luminosity, compact size, and known spectral irradiance. Placing the light source approximately 5 cm from the reaction ensured efficient photo-excitation.

Dehydrogenation of Alkanes and Alcohols

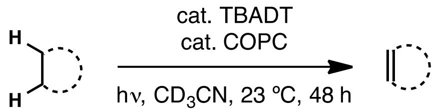

An oven-dried 17 × 60 mm (8 mL) borosilicate vial (for transmittance characteristics see section IV) was equipped with a magnetic stir bar, TBADT[21] (0.004–0.03 equiv), and COPC[22] (0.004–0.015 equiv). Following the addition of methyl acetate (3-4 drops) and CD_3CN (1.1 mL, ~0.65 M) the vial was fitted with a silicone septa screw cap and sparged with argon for 10 minutes. The substrate (0.65 mmol, 1 equiv, previously sparged with Ar) was added by syringe and stirred vigorously away from UV light sources. A t=0 aliquot was then removed using a syringe for calibrating the internal standard and the vial was sealed with parafilm and placed approximately 5 cm from a 26 W compact fluorescent light bulb.[23] Successful reactions changed colour from clear, pale yellow solutions to dark blue solutions within 1 h of the start of irradiation. After 48 hours, hydrogen production was confirmed by headspace GC/TCD analysis. The alkene production was then assayed using the method described below. If headspace analysis was not required, it is prudent to note that breakage of the vial seal was accompanied by the release of pressure, corresponding to the generated hydrogen.

Alternatively, if hydrogen production was to be quantified, the reaction was set up analogously to above save no internal standard was included and no aliquots were removed from the reaction mixture. After the specified time of irradiation, the volume of evolved gas was measured through the equilibration of the reaction headspace pressure with atmospheric pressure using a gas syringe. As hydrogen is the only new component of the headspace following the reaction, the excess volume is attributed solely to hydrogen evolution. Following the measurement of hydrogen, the amount of alkene was determined using the 1,3,5-trimethoxybenzene method described below.

The moles of evolved hydrogen was found to correspond to the moles of produced alkene in a 1:1 ratio.

The dehydrogenation of alcohols was carried out analogously to that of alkanes save that the concentration of substrate was reduced from 0.65M to 0.5M.

Spectral Output of Lamp

The spectral output of the Exo-Terra Reptile 150 lamp can be found online at:

http://www.exo-terra.com/en/products/reptile_uvb150.php

Transmittance of Reaction Glassware

All photoreactions were carried out in Fisherbrand 03-339-21D 8 mL vials as described in the procedure. These vials are made from code 7800 pharmaceutical glass, a type 1 class B borosilicate glass of a thickness of ~1 mm. The glass transmits ~80% of incident 325 nm light and over 90% for 360 nm and greater. As decatungstate excitation occurs above 325 nm, we are confident that sufficient luminous flux at the necessary wavelength occurs to efficiently excite the TBADT. While cobaloximes are known to undergo some photochemistry in the same frequency regime, it appears that this reactivity is of less significance (*vide infra*).

A transmittance graph for code 7800 pharmaceutical glass is provided by Corning online:

http://csmedia2.corning.com/LifeSciences//media/pdf/Description_of_%20Code_7800.pdf

Optimization Studies

Study of the dehydrogenation of cyclooctane using various catalyst loadings showed that the reaction required more than one day to achieve a maximum amount of product (Supplementary Table 3). However, irradiation for more than 48 h led to the precipitation of solid (potentially polymeric side products) and did not meaningfully increase the observed yield of cyclooctene.

Additionally, extended irradiation times (particularly at higher TBADT loadings) were frequently accompanied by the competitive decomposition of the tetrabutylammonium counterion as described by Yamase.[24] This result is unsurprising as four equivalents of this competing substrate are introduced for every one decatungstate molecule, quickly amounting to a significant concentration at higher decatungstate loadings. Attempts to use concentrations above 3 mol% TBADT were also hampered by the limited solubility of the complex in acetonitrile.

Study of the alcohol dehydrogenation showed many of the same results as observed for the alkane dehydrogenation control reactions (Supplementary Table 4). Entry 8 was performed under ambient atmosphere, showing that TBADT-catalyzed oxidation of alcohols via adventitious oxygen is slower than the dual catalytic reaction.

Cobalt Catalyst Screen

Cobalt cocatalysts[25,26,27] were screened using the reaction conditions described above using 1.5 mol% TBADT and 0.75 mol% cobalt cocatalyst. Results reported are after 48 hours of irradiation, as %yield (cooperative TON) in Supplementary Fig. 32.

The Choice of Solvent

Very few solvents (acetonitrile, acetone, and water) are compatible with decatungstate photocatalysis;[28] many common solvents serve as competitive substrates for the dehydrogenation reaction and so were not explored extensively. Additionally, the reactivity of cobaloximes is well studied in acetonitrile, leading us to focus our investigation to conditions using this solvent.

Method For Determining *in situ* yields

An initial t = 0 aliquot (100 µL) of the reaction mixture is diluted to 500 µL using CD_3CN and aerated via vigorous shaking. Integration of the characteristic ^{1}H NMR substrate peak for each alkane (δ 1.54 for cyclooctane, 1.51 for cyclopentane, 1.44 for cyclohexane, and 2.02 for ethyl isovalerate) followed by normalization to the correct number of protons (16, 10, 12, and 1 respectively) allows for the integration of the well-resolved methyl acetate O–CH_3 peak (δ 3.59) to serve as the calibrated standard. Following the specified irradiation time, additional aliquots may be removed and prepared using the above procedure to acquire a spectrum. The O–CH_3 peak of the standard is then integrated to the same value as measured in the t=0 and both the starting material (see above) and product (δ 5.62 for cyclooctene; 5.74 for cyclopentene and strongly coupled doublets for cyclopentadiene centered at 6.51; 5.66 for cyclohexene, multiplets centered at 5.80 and 5.89 for 1,3-cyclohexadiene and singlet 7.37 for benzene; doublet at 4.85 for ethyl isovalerate). Additionally, tetrabutylammonium decomposition products (1-butene and tri-*n*-butylamine) were observed in reactions using higher TBADT loadings. The identity of these compounds was confirmed via addition of authentic product followed by increase of the respective peak intensities in the spectrum.

The high reactivity of TBADT precludes the use of many common internal standards as these can serve as competitive substrates for the initial HAT. Methyl acetate was chosen due to the very low reactivity of hydrogen atoms alpha to ketones and ester oxygens under TBADT photocatalysis[29] in addition to the paucity of competing peaks in the 3.5–4.0 window of the ^{1}H NMR spectrum.

Alternatively, the reaction could be quenched by the addition of ambient atmosphere and a known amount of 1,3,5-trimethoxybenzene was added to the solution. Once the standard was dissolved, an NMR was taken and the substrate was quantified by the relevant integration of a characteristic peak (see above) against either the peak at 6.09 (s, 3H) or 3.74 (s, 9H). This method provided results in agreement with the methyl acetate protocol described above.

UV-Vis Continuous Photolysis Experiences

All UV-Vis continuous photolysis experiments were caried out under standard reaction conditions save a ~3 fold dilution. All spectra are zeroed with respect to a straight-acetonitrile blank. Reaction mixtures were prepared under ambient atmosphere and transferred to a gas-tight quartz cuvette with septum that was then sparged with argon for 10 minutes. Following the acquisition of the time 0 time point, the cuvette was irradiated at a distance of ~5 cm from an Exo-Terra Reptile 150 lamp with subsequent measurements taken at the indicated times; the reaction was thoroughly mixed prior to each measurement through repeated inversion.

At higher absorbance values, the line-emission from the deuterium arc lamp, Dα, becomes a visible artifact in the spectrum as a sharp peak at 656 nm.

The reaction mixture was aerated following the final time point through exposure to ambient atmosphere followed by repeated inversion.

Supplementary References

[1] Renneke, R.F., Pasquali, M. & Hill, C.L. *J. Am. Chem. Soc.* **1990**, *112*, 6585.
[2] Dempsey, J.L., Brunschwig, B.S., Winkler, J.R. & Gray, H.B. Hydrogen Evolution Catalyzed by Cobaloximes. *Acc. Chem. Res.* **2009**, *42*, 1995.
[3] Paczesniak, T.; Bloniarz, P. Rydel, K.; Sobkowiak, A. *Electroanalysis* **2007**, *19*, 945.
[4] Li, Y.; Zhou, X.-T.; Ji, H.-B. *Cat. Commun.* **2012**, *27*, 169.
[5] Pavlishchuk, V.V.; Addison, A.W. *Inorg. Chim. Acta* **2000**, *298*, 97.
[6] Tanielian, C.; Seghrouchni, R.; Schweitzer, C. *J. Phys. Chem. A.* **2003**, *107*, 1102.
[7] Li, G., Pulling, M.E., Estes, D.P. & Norton, J.R. Cobaloxime-Mediated Radical Cyclization under H_2: Evidence for Formation of a Co–H Bond and Kinetics of H• Transfer. *J. Am. Chem. Soc.* **2012**, *134*, 14662.
[8] Lazarides, T.; McCormic, T.; Du, P.; Luo, G.; Lindley, B.; Eisenberg, R. *J. Am. Chem. Soc.* **2009**, *131*, 9192.
[9] Kahnt, A.; Peuntinger, K.; Dammann, C.; Drewello, T.; Hermann, R.; Naumov, S.; Abel, B.; Guldi, D.M. *J. Phys. Chem. A.* **2014**, *118*, 4382.
[10] Lacy, D.C.; Roberts, G.M.; Peters, J.C. *J. Am. Chem. Soc.* **2015**, *137*, 4860.
[11] Dempsey, J.L. Hydrogen Evolution Catalyzed By Cobaloximes. Ph.D. Dissertation, California Institute of Technology, Pasadena, CA, 2011.
[12] Tsou, T.–T.; Loots, M.; Halpern, J. *J. Am. Chem. Soc.* **1981**, *104*, 623.
[13] Pierik, B.; Masclee, D.; Vollmerhaus, R.; van Herk, A.; German, A. L. In Free Radical Polymerization. Kinetics and Mechanism, Macromolecular Symposia Series; Buback, M., Ed.; Wiley- VCH: Weinheim, 3rd IUPAC-Sponsored International Sympo- siumonFreeRadicalPolymerization: KineticsAndMechanism, Il Chioccho/Lucca, Italy, June 3-8, 2001.

[14] Hu, X.; Brunschwig, B.S.; Peters, J.C. *J. Am. Chem. Soc.* **2006**, *129*, 8988.
[15] Tian, Z.; Fattahi, A.; Lis, L.; Kass, S.R. *J. Am. Chem. Soc.* **2006**, *128*, 17087.
[16] Zhang, X.-M. *J. Org. Chem.* **1998**, *63*, 1872.

[17] Kalyanasundaram, K.; Kiwi, J.; Gratzel, M. *Helv. Chim. Acta* **1978**, *61*, 2720.
[18] Perrin, D. D.; Armarego, W. L. F. *Purification of Laboratory Chemicals*; 3rd ed., Pergamon Press, Oxford, 1988.
[19] Pangborn, A. B.; Giardello, M. A.; Grubbs, R. H.; Rosen, R. K.; Timmers, F. J. *Organometallics,* **1996**, *15*, 1518.
[20] Still, W. C.; Kahn, M.; Mitra, A. J. *J. Org. Chem.* **1978**, *43*, 2923.
[21] TBADT was prepared according to Protti, S., Ravelli, D., Fagnoni, M., Albini, *Chem. Commun.* **2009**, 7351.
[22] COPC is commercially available from Aldrich (CAS: 23295-32-1; Product ID: 341630); alternatively, it can be easily prepared as described in Schrauzer, G.N. *Inorg. Synth.* **1968**, *11*, 61.
[23] ExoTerra Reptile UVB 150 26W CFL bulb (Product ID: PT2189)
[24] Yamase, T., Takabayashi, N. & Kaji, M. Solution Chemistry of Tetrakis(tetrabutylammonium) Decatungstate(VI) and Catalytic Hydrogen Evolution from Alcohols. *J. Chem. Soc. Dalton Trans.***1984,** 793.
[25] COBF was prepared as described in Bakac, A.; Espenson, J.H. *J. Am. Chem. Soc.* **1984**, *106*, 5197. COBF can also be purchased from strem (CAS: 26220-72-4)
[26] $Co(OAc)_2•4(H_2O)$ was purchased from Strem (CAS: 6147-53-1)
[27]$Co(acac)_2•x(H_2O)$ was purchased from Strem (CAS: 123334-29-2)
[28] Tanielian, C.; Cougnon, F.; Seghrouchni, R. *J. Mol. Catal. A.* **2006**, *262*, 164.
[29] Okada, M.; Fukuyama, T.; Yamada, K.; Ryu, I.; Ravelli, D.; Fagnoni, M. *Chem. Sci.* **2014**, *5*, 2893.
